# Oligodendrocyte precursor cells ingest axons in the mouse neocortex

**DOI:** 10.1073/pnas.2202580119

**Published:** 2022-11-23

**Authors:** JoAnn Buchanan, Leila Elabbady, Forrest Collman, Nikolas L. Jorstad, Trygve E. Bakken, Carolyn Ott, Jenna Glatzer, Adam A. Bleckert, Agnes L. Bodor, Derrick Brittain, Daniel J. Bumbarger, Gayathri Mahalingam, Sharmishtaa Seshamani, Casey Schneider-Mizell, Marc M. Takeno, Russel Torres, Wenjing Yin, Rebecca D. Hodge, Manuel Castro, Sven Dorkenwald, Dodam Ih, Chris S. Jordan, Nico Kemnitz, Kisuk Lee, Ran Lu, Thomas Macrina, Shang Mu, Sergiy Popovych, William M. Silversmith, Ignacio Tartavull, Nicholas L. Turner, Alyssa M. Wilson, William Wong, Jingpeng Wu, Aleksandar Zlateski, Jonathan Zung, Jennifer Lippincott-Schwartz, Ed S. Lein, H. Sebastian Seung, Dwight E. Bergles, R. Clay Reid, Nuno Maçarico da Costa

**Affiliations:** ^a^Allen Institute for Brain Sciences, Seattle, WA 98109; ^b^Janelia Research Campus, Howard Hughes Medical Institute, Ashburn, VA 20147; ^c^The Solomon H. Snyder Department of Neuroscience, John Hopkins University School of Medicine, Baltimore, MD 21205; ^d^Princeton Neuroscience Institute, Princeton University, Princeton, NJ 08540; ^e^Computer Science Department, Princeton University, Princeton, NJ 08554; ^f^Johns Hopkins Kavli Neuroscience Discovery Institute, Baltimore, MD 21205

**Keywords:** oligodendrocyte precursor cells, engulfment, phagolysosomes, axonal pruning

## Abstract

Neurons in the developing brain undergo extensive structural refinement as nascent circuits adopt their mature form. This physical transformation of neurons is facilitated by the engulfment and degradation of axonal branches and synapses by surrounding glial cells, including microglia and astrocytes. However, the small size of phagocytic organelles and the complex, highly ramified morphology of glia have made it difficult to define the contribution of these and other glial cell types to this crucial process. Here, we used large-scale, serial section transmission electron microscopy (TEM) with computational volume segmentation to reconstruct the complete 3D morphologies of distinct glial types in the mouse visual cortex, providing unprecedented resolution of their morphology and composition. Unexpectedly, we discovered that the fine processes of oligodendrocyte precursor cells (OPCs), a population of abundant, highly dynamic glial progenitors, frequently surrounded small branches of axons. Numerous phagosomes and phagolysosomes (PLs) containing fragments of axons and vesicular structures were present inside their processes, suggesting that OPCs engage in axon pruning. Single-nucleus RNA sequencing from the developing mouse cortex revealed that OPCs express key phagocytic genes at this stage, as well as neuronal transcripts, consistent with active axon engulfment. Although microglia are thought to be responsible for the majority of synaptic pruning and structural refinement, PLs were ten times more abundant in OPCs than in microglia at this stage, and these structures were markedly less abundant in newly generated oligodendrocytes, suggesting that OPCs contribute substantially to the refinement of neuronal circuits during cortical development.

## Significance

Oligodendrocyte precursor cells (OPCs) are a population of glia cells that tile across the brain and retain the ability to proliferate. They can become myelinating oligodendrocytes or remain in the precursor state. An unresolved question has been what other functions OPCs might carry out. Using high-throughput transmission electron microscopy and dense 3D reconstructions, we were able to visualize evidence of ingestion and breakdown of axons by OPCs. Analysis of the abundant tertiary lysosomes or phagolysosomes (PLs) in OPC branches confirmed the presence of 40-nm vesicular structures within their chambers. Furthermore, analysis of PLs in microglia demonstrated that their numbers were significantly lower and rarely contained vesicles. This study provides evidence of engulfment and pruning by OPCs.

Oligodendrocyte precursor cells (OPCs) emerge from several germinal zones in late prenatal development following the sequential generation of neurons and astrocytes, migrate into the expanding cortex, and then proliferate to establish a grid-like distribution, with individual cells occupying distinct territories. Genetic fate tracing and time lapse imaging in vivo have demonstrated that these progenitors play a critical role in generating oligodendrocytes and thus myelin throughout the central nervous system (CNS) (1). However, OPCs are present in some cortical regions weeks before oligodendrogenesis begins and extend highly ramified, dynamic processes that contact developing neurons. These progenitors express a diverse set of neurotransmitter receptors and form direct, functional synapses with excitatory and inhibitory neurons (2, 3). Such features have traditionally been viewed through the perspective of oligodendrogenesis (4), as other roles for these ubiquitous glial cells have not been clearly established (2, 3).

**Fig. 1.**
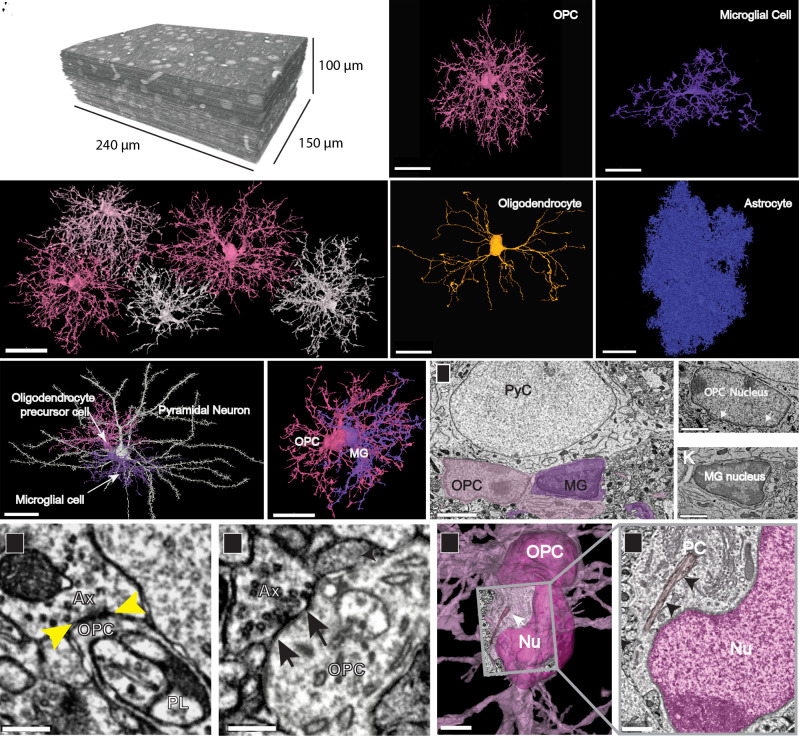
Distinct structural features of OPCs in the developing visual cortex. (*A*) TEM reconstruction of 100-µm^3^ volume of layer 2/3 mouse visual cortex (P36). (*B*) 3D reconstructions of a subset of OPCs in P36 dataset, showing discrete territories and tiling. (Scale bar, 30 µm.) (*C*) 3D rendering of an OPC from P36 dataset showing extensive ramifications emanating from the cell soma. (*D*) 3D rendering of a microglial cell shows its thicker, less branched processes and elongated and flattened soma. (*E*) 3D rendering of a mature myelinating oligodendrocyte in P36 dataset has a smooth and ovoid-shaped soma. Note, only the soma and cytoplasmic processes without myelin sheaths are shown. (*F*) 3D rendering of an astrocyte in P36 dataset showing its densely packed cytoplasmic protrusions. (Scale bars, *C–F*, 20 µm.) (*G*) 3D rendered pyramidal neuron (white) with an OPC (pink) and a microglial cell (MG, purple) both in satellite positions. (Scale bar, 30 µm.) (Movie S1) (*H*) 3D rendering of the same two glial cells in (*G*) shows their close association and intermingling of branches. (Scale bar, 20 µm.) (*I*) Ultrathin section slice through an OPC soma (pink) and microglial cell soma (purple). (Scale bar, 3 μm.) (*J*) Ultrathin section slice of an OPC nucleus with dense rim of heterochromatin and ruffled edge (white arrows). (Scale bar, 1.5 μm.) (*K*) Ultrathin section slice of a microglial nucleus showing its dense heterochromatin throughout. (Scale bar, 1.5 μm.) (*L* and *M*) Axons (Ax) making synaptic contacts (yellow and black arrows) with OPC processes. A phagolysosome (PL) is nearby in *L*. (Scale bar, 300 nm.) (*N*) The soma of an OPC (deep pink) bears a primary cilium (white arrow) adjacent to the nucleus (Nu). (Scale bar, 3 µm.) (*O*) Ultrathin slice of the boxed area in *N* showing the primary cilium (PC) (black arrows) close to the OPC nucleus (Nu, dark pink). (Scale bar, 750 nm.)

Our knowledge about the structure and function of OPCs has been severely limited by incomplete ultrastructural information, because their fine, highly branched processes are difficult to unambiguously recognize in electron microscopy (EM) studies without complete reconstructions (5) to connect them to identifiable somata. Modern computational volume EM methods offer an unprecedented opportunity to expand our understanding of brain ultrastructure (5–7), particularly for morphologically complex, highly dynamic glial cells like OPCs. Here, we used two densely segmented and reconstructed datasets of the mouse visual cortex, aged postnatal day (P)36 (Fig. 1*A*) and P54, to perform a detailed morphometric analysis and quantification of the anatomical features of somata, processes, and organelles therein of OPCs at this highly dynamic phase of neocortical maturation to help define their roles in the developing brain.

## Structural Features of OPCs Revealed by Large Scale Serial TEM

Past EM ultrastructural studies defined several common morphological characteristics of OPCs, such as their oblong nucleus containing low heterochromatin and presence of centrioles in their cytoplasm (8), consistent with their proliferative progenitor state (9). Originally referred to as a multipotential type of glia (10), and often termed NG2 cells (11, 12) in reference to their expression of the proteoglycan NG2 (Cspg4), early EM investigations often focused on their cellular responses to injury and the close association of their processes with synapses and degenerating nerve fibers (11, 13). More recent studies in naïve animals indicate that OPC processes form direct synapses with axons and that OPCs are sometimes associated with nodes of Ranvier (2, 14); however, quantitative analysis of these structural features has been difficult due to the limited sampling of the whole volume of these cells (2, 11, 13, 14).

**Fig. 2.**
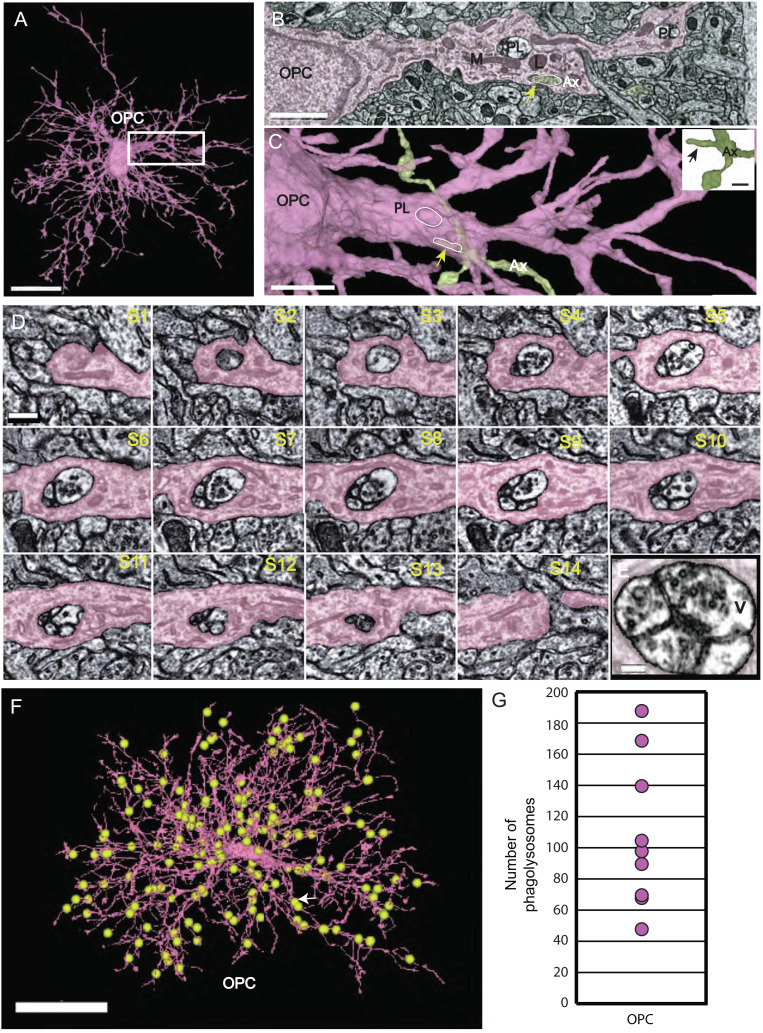
Phagolysosomes (PLs) are abundant in OPC processes and contain vesicles. (*A*) 3D rendering of OPC with numerous ramified branches. Boxed area same as micrograph shown in (*B*) (Scale bar 15µm.) (*B*) OPC branch contains PLs, lysosomes (L), and mitochondria (M), and an ingested axon (yellow arrow) (Ax). (Scale bar 1.5 µm.) (*C*) 3D rendering of same OPC in (*B*) in pink and axon (Ax) (green) arrow (yellow) points to piece of ingested axon outlined in white and PL (white oval). (Scale bar 2 µm.) *Inset* shows 3D rendering of the same green axon (Ax) bouton with small, ingested protrusion outlined in slice view (yellow arrow). (Scale bar 500 nm.) (*D*) Twelve ultrathin 40-nm serial sections through a phagolysosome show the distribution of 40-nm vesicles within the organelle. Nine of the twelve sections have vesicles inside the phagolysosome. (Scale bar 300 nm.) (*E*) The last panel shows high magnification of vesicles inside phagolysosome. (Scale bar 150 nm.) (*F*) 3D reconstruction of an OPC from the P36 dataset. Yellow spheres represent manual annotations of the 187 PLs found in this OPC. White arrow points to location of PL in serial section Fig. 4*D*. (Scale bar, 20 μm.) (original data in http://microns-explorer.org/phagolysosomes/opc). (*G*) Plot showing the number of PLs in nine OPCs in P36 dataset. See Movie S2.

The two large-volume, serial TEM datasets were collected from the visual cortex of young transgenic mice aged P36 (Fig. 1*A*) and P54 to enable correlative connectomic analysis after functional in vivo imaging (15, 16). We used these TEM datasets to carry out a detailed structural analysis of different glial cell types within this region, enabling quantitative analysis of their ultrastructural features and 3D renderings. Four distinct classes of glia were present within this region of the cortex: OPCs (Fig. 1 *B* and *C*), microglia (Fig. 1*D*), oligodendrocytes (Fig.1*E*), and astrocytes (Fig. 1*F*). OPCs exhibited a ramified form with 15 to 17 highly branched processes extending up to 50 µm radially from the soma (Fig. 1 *B-C, G-H*) and *SI Appendix*, Fig. S1 *A*–*H*), which contained numerous filopodia on their tips. 3D renderings of adjacent OPCs revealed that these cells, like astrocytes and microglia (17, 18), were distributed in a grid-like organization with little overlap between territories of neighboring cells (Fig. 1*B* and *SI Appendix*, Fig. S2), and contacted synapses (19, 20). OPC somata were frequently in a satellite position, like those of microglia and oligodendrocytes (21, 22) (Fig. 1*G* and *SI Appendix*, Fig. S1 *A*, *B*, *D*, *F*, *G*, and *H* and Movie S1), and were remarkably variable in both size and shape, ranging from elongated or bean shaped to smooth or irregular with a rough surface (Fig. 1*B* and *SI Appendix*, Fig. S1 *A*–*H*). OPCs were readily distinguished from other glial cell types by these ultrastructural features, as well as having: 1) larger nuclei that were elongated and contained less heterochromatin than those of microglia (Fig. 1 *I*–*K*); 2) processes that were longer and smoother compared to those of microglia or astrocytes (Fig. 1 *C*, *D*, and *F*); 3) axons that formed synaptic contacts with their processes (Fig. 1 *L* and *M*); 4) cytoplasm that was more electron lucent than that of microglia and devoid of glycogen granules that are numerous in astrocytes (Fig. 1*M*); and 5) the presence of primary cilia, which were not found on microglia, premyelinating or mature oligodendrocytes (Fig. 1 *N* and *O* and *SI Appendix*, Fig. S1 *A*–*I*). Primary cilia were found on all OPCs in both datasets and this feature helped distinguish them from premyelinating and mature oligodendrocytes (23). The size of the datasets combined with the TEM resolution and dense segmentation, shows the anatomy and structure of the OPCs at both low and high scales. One can investigate the fine ultrastructure of these cells at the high-resolution offered by TEM as well as zoomed out to a lower scale and see most of the arbor of the segmented OPC (Figs. 1 *C* and 2 *A* and *F*).

## OPC Processes Contain Numerous Lysosomes and Phagolysosomes

Previous in vivo imaging experiments revealed the dynamic nature of OPC processes, which exhibit continuous branch remodeling as they migrate through gray matter, making transient interactions with various cellular constituents (24). Analysis of OPC processes in TEM images from this study aided by whole-cell reconstructions, revealed that these processes (Fig. 2*A* and *SI Appendix*, Fig. S3*A*) frequently contacted axons (Fig. 2 *B* and *C* and *SI Appendix*, Fig. S2 *B* and *C*). Moreover, the cytoplasm of these processes often contained numerous membrane-bound organelles, most prominently phagosomes, lysosomes (Fig. 2*B*), and digestive organelles known as phagolysosomes (PLs) (Fig. 2 *B* and *C* and *SI Appendix*, Fig. S2 *B* and *C* and Movie S2), suggesting that OPCs at this age engage in phagocytosis.

The process of phagocytosis begins with the recognition of a target, followed by phagosome formation (engulfment), late phagosome maturation, and finally fusion of the phagosome with a lysosome, forming the highly acidic phagolysosome (25). If OPCs engage in engulfment of an external material at this age, various stages of phagocytosis should be visible. Accordingly, we found in both TEM volumes, completely internalized, membrane-delimited structures resembling phagosomes and material partially engulfed within OPC cytoplasm, with some connection remaining to the external target, usually axons (Fig. 2 *B* and *C*). In addition, lysosomes were also abundant in OPC processes, visible as electron-dense intracellular organelles measuring ~300–500 nm in diameter (Fig. 2*B* and Movie S2) that were readily distinguishable from the larger multichambered PLs measuring 500–750 nm in diameter (Fig. 2*B*). Seldom visualized in large numbers by EM, PLs represent the last step of phagocytosis, and constitute a highly acidic compartment needed to break down ingested elements (25, 26). Although some of the material inside the OPC PLs was unidentifiable cellular debris, these organelles frequently contained clusters of small (~40 nm) clear vesicles (Fig. 2*D*). These were numerous within the phagolysosome, as shown in serial sections and at a high magnification (Fig. 2 *D* and *E*). Because some OPCs were contained with the serial volume, it was possible to map the distribution of PLs across all of their processes and somata (Fig. 2 *F* and *G* and *SI Appendix*, Fig. S1 *A*–*H* and *I*), revealing that PLs were widely distributed in all 18 OPCs that were analyzed in both datasets and particularly enriched at near the tips of processes. Quantitative analysis from reconstructed OPCs revealed that 38 ± 14% of the PLs contained vesicles at P36 (mean ± standard deviation, range 22–58%, n = 9 OPCs), indicating that this is a widespread phenomenon. Since the vesicles found inside the PLs were of the same size as the ones found in synaptic boutons, we asked if OPCs were engulfing portions of nearby axons and associated synapses.

## OPCs Engulf Axons in the Mouse Cortex

**Fig. 3.**
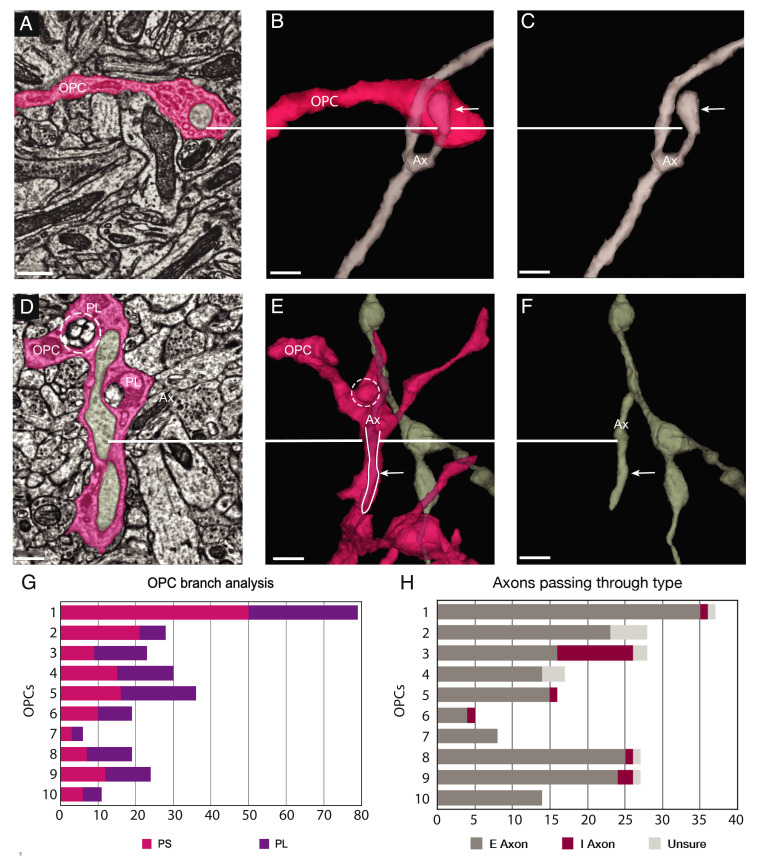
Axon engulfment by OPCs. (*A*) OPC process in pink ingests an excitatory axon bouton (gray) at its tip. (Scale bar, 500 nm.) (*B*) 3D reconstruction reveals a small excitatory axon fragment (Ax) encapsulated within the OPC (pink) at the tip (white line, arrow). (Scale bar, 500 nm.) (*C*) 3D reconstruction shows the same axon (Ax)(arrow) without OPC surrounding it. The bouton tip is visible. (Scale bar, 500 nm.) (*D*) Ultrathin section slice of a large section of an inhibitory axon (Ax) collateral branch (white line) in gray ingested within the cytoplasm of the OPC (pink). Two PL (one in dashed circle) are adjacent to the ingested axon. (Scale bar, 500 nm.) (*E)* 3D rendered axon collateral branch encapsulated within the cytoplasm of the OPC (arrow, white line and outline) and phagolysosome within the cytoplasm (dashed circle). (Scale bar, 1μm.) (*F*) 3D reconstruction of the axon (Ax) in gray shows encapsulated branch (white line, arrow) without the surrounding OPC. (Scale bar, 1μm.) (*G*) Bar graph of ten individual isolated main branches (one each) of ten OPC cells were analyzed for ingestion events and categorized as either phagosome (PS)(pink) or phagolysosome (PL)(purple). (*H)* Bar graph of the same ten OPCs showing axons partially ingested typed as excitatory (E), inhibitory (I) or unsure (U). Excitatory axons were the most prevalent type ingested at 85.9% versus 7.7% for inhibitory.

The appearance of clusters of 40 nm clear vesicles inside a subset of PLs in every OPC (*SI Appendix*, Figs. S1 *A*–*I* and S3) suggests the possibility of presynaptic terminals or portions of axons containing terminals are engulfed by these cells. Analysis of the 3D reconstructions revealed that indeed, small cytoplasmic protrusions and small branches of axons were often surrounded by OPC processes (Fig. 3 *A*–*F* and *SI Appendix*, Figs. S1 *A*–*H*, S4, and S5 and Movie S3). The complex anatomy of the branches and the need to annotate and quantify the ingestions made this analysis particularly challenging. To simplify the analysis, ten individual main branches from ten distinct OPCs in the P36 dataset were surveyed for ingested materials (See *SI Appendix*, Fig. S6 for an example). First, we categorized the ingestions as phagosomes (PS) or (PLs), and found they were present in every OPC in variable amounts (range 5–27 PS and 6–50 PL) (Fig. 3*G*). Next, we identified on the same ten OPC branch locations where axon engulfment was occurring (Fig. 3*H* and *SI Appendix*, Table S1). In these types of ingestions, there was no evidence of injury or destruction to the axon, only local contact with OPC cytoplasmic processes that encased the collateral or terminal region of the axon but did not fully encapsulate it. Two types of axon engulfment were observed: either small bulb like axonal buds ranging from 300–500 nm^3^ or small branches ranging from 1–4 μm (*SI Appendix*, Table S1) surrounded by an OPC process, in which the engulfed material remained connected to the parent axon (Fig. 3 *B* and *E* and *SI Appendix*, Figs. S5 and S6 and Movie S3).

OPCs form synapses with both excitatory and inhibitory neurons in the developing cortex (2, 3) and axons from both neuron classes are targeted for myelination in the cortex (27, 28), indicating that OPCs and their subsequent later developmental stages interact with distinct cell classes. To determine if axon engulfment is specific to one class of neurons, we traced 195 axons partially engulfed within the isolated branches of ten OPCs back to the main axon and examined their synaptic boutons, classifying them as excitatory or inhibitory based on ultrastructural synaptic morphology (*SI Appendix*, Fig. S7). Since the volume did not contain entire neurons, we were unable to identify the cell type of axonal origin and only whether they were excitatory (E) or inhibitory (I), based on anatomical features of the pre- and postsynaptic membranes, specifically their electron density. Because the engulfed axons usually formed multiple synapses, we used the nature of the postsynaptic structure as further confirmation of our classifications, since excitatory axons make most of their synapses with spines while inhibitory axons make most of their synapses with somata and dendritic shafts.

This analysis showed the predominant type of axons within the ten isolated branches of OPCs were excitatory (85.9 %) or inhibitory (7.7%) (Fig. 3*H* and *SI Appendix*, Figs. S5 *A*–*H* and S6) with 6.3% undetermined. Approximately 7.7% of the identifiable ingested axons were inhibitory, suggesting that OPCs were mainly removing portions of excitatory axons in this dataset. Although interneurons comprise 10–20% of all neurons in the neocortex, the association of OPCs with interneurons may be lower because the P36 dataset was restricted to layer 2/3 where the interneuron density is lower. Overall, the numbers of all types of ingestions were variable among the ten OPC branches, possibly indicative of the phagocytic capability of the individual cells. The association of OPCs with axon fragments and small axon branches was observed in all OPCs from both P36 and P54 datasets, indicating that axon engulfment is a conserved function of OPCs during this period. Such associations may represent the predecessor to large-scale axonal pruning and suggest that small segments of axon collaterals are targeted for removal by OPCs.

## Phagolysosomes are More Abundant in OPCs than Microglia

**Fig. 4.**
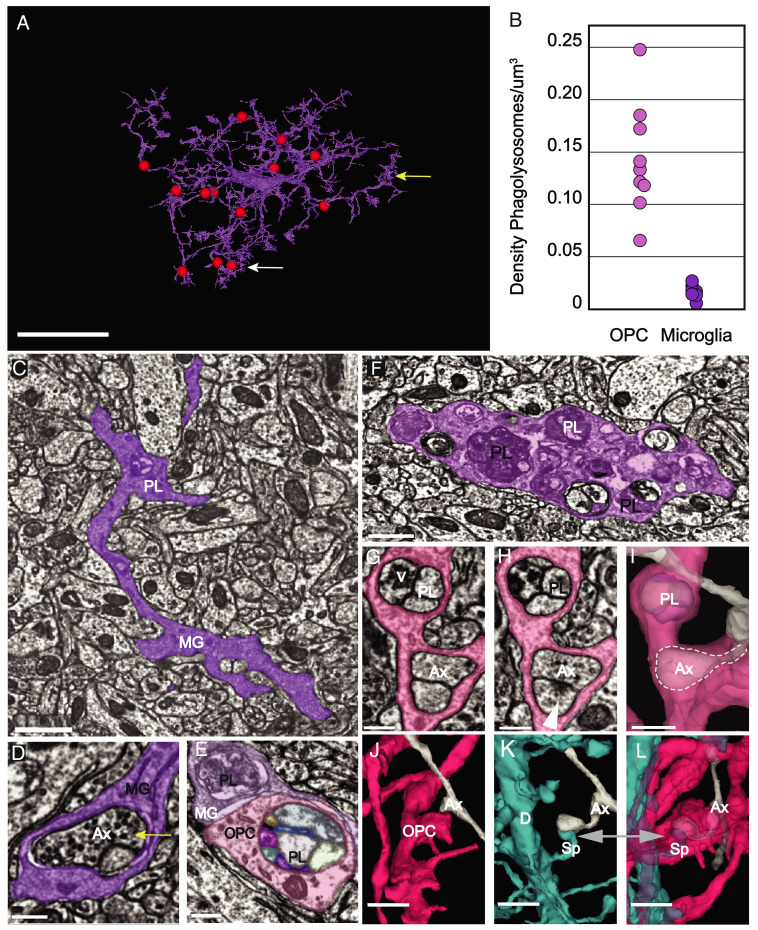
PLs are more abundant in OPCs than microglia. (*A*) 3D rendering of a microglial cell from the P36 dataset. Red spheres represent manual annotations of the 13 PLs present in this cell. White arrow points to the location of the PL shown in 4*C*. (Scale bar, 10 μm.) (original data in http://microns-explorer.org/phagolysosomes/microglia. (*B*) Comparison of density of PLs at P36 between OPCs and microglia. Density was calculated including the volume of the soma region. (*C*) Microglial cell branch (MG) of cell shown in (*A*) in purple shows a phagolysosome (PL) (white arrow also shown in 4*A*) in its dense cytoplasm. (Scale bar, 1 µm.) (*D*) A synapse is ingested within the microglia branch. Its position in the cell is marked by the yellow arrow also shown in 4*A*. Vesicles in the presynaptic axon (Ax) are visible. (Scale bar 300 nm.) (*E*) A microglial (MG) branch (purple) from P36 dataset contacts and OPC branch in pink with PLs in both cells. (Scale bar, 300 nm.) (*F*) A portion of a microglial branch shows the cytoplasm congested with numerous PLs. (Scale bar, 750 nm.) *(E)* A microglial (MG) branch (purple) from P36 dataset contacts and OPC branch in pink with PLs in both cells show their different morphologies. (Scale bar, 300 nm.) *(G)* OPC process contains a phagolysosome with vesicles (V) and engulfed axon (Ax). (Scale bar 300 nm.) (*H*) A different slice of the same process shows the phagolysosome (PL) and postsynaptic density (arrow) of a synapse onto the encapsulated axon, which remains attached to its parent. (Scale bar 300 nm.) (*I*) 3D rendering of the same process shows both the phagolysosome (PL) and the encapsulated axon collateral within the OPC cytoplasm(dashed outline). (Scale bar 500 nm.) (*J*) 3D rendering of the same OPC in (*G*) and axon (Ax) in gray. (*K*) 3D rendering of the dendrite (D) in blue shows the spine (Sp) in blue synapsing onto the same axon (Ax) in (*J*). (*L*) The spine (Sp) and collateral axon branch (Ax) remain attached to their cells of origin but are fully surrounded by OPC cytoplasm. (Scale bars, (*J–L*) 1 μm.) See Movie S4.

The presence of abundant PLs in OPCs was unexpected, as these cells have mostly been seen as precursors to oligodendrocytes. Moreover, most structural pruning of neurons is thought to be mediated primarily by microglia (29, 30). To determine the relative abundance of PLs in these two glial cell types, we quantified PLs in complete 3D reconstructions of nine OPCs and nine microglia in the same volume of the P36 dataset. Examples of those annotations of one OPC (Fig. 2*F*) and one microglia from the P36 dataset is shown in Fig. 4*A*. This analysis revealed that PLs were significantly more abundant in OPCs than microglia at this age (PLs/cell: OPCs, 107 ± 48; microglia, 9 ± 3; Student’s *t* test, *P* < 0.001, n = 9). Although some OPCs are larger than microglia at this age, the density of PLs was still significantly higher in OPCs when normalized to cell volume (Fig. 4*B*) (PL density: OPCs, 0.143 ± 0.05 PLs/µm^3^; microglia, 0.03 ± 0.01 PLs/µm^3^; *P* < 0.001, Student’s *t* test, n = 9); suggesting that OPCs are more actively engaged in neuronal process engulfment than microglia at this age.

Microglia have been shown to engulf diverse materials, including cell corpses and myelin fragments (31, 32). Although microglial PLs were observed within the branches and appeared to contain unidentifiable electron-dense material and an occasional synapse (Fig. 4 *C* and *D*) they rarely contained 40-nm vesicles like the OPC PLs (average: 18 ± 15% of PLs at P36; 14 ± 18% at P54) (Fig. 4 *E* and *F*). They were also more difficult to find and identify because of their variable morphologies and sparse occurrences, unlike those of OPCs (Fig. 4 *E* and *F*). Although we often observed vesicles inside PLs, events where we observed postsynaptic densities, and therefore an entire synapse contained within an OPC process (e.g., Fig. 4 *G*–*L*) were infrequent, (Fig. 4 *G–I*, 3*D* view Fig. 4 *J–L* and Movie S4). Occasionally, we observed dendritic spines encompassed within the OPC branches, but this was also rare – in the P36 dataset, five excitatory synapses and six dendritic spines were found in the ten OPC branches. Like the axonal collateral branches, these encapsulations were connected to their parent axon or dendrites and did not appear to be damaged or unhealthy. The scarcity of 40-nm vesicles inside microglia PLs and their heterogeneity in both size and contents compared to those of OPCs suggest that these two glial cell types recognize and engulf distinct cellular elements.

To further support the finding of the prevalence of these organelles in OPC branches, OPCs isolated from the visual cortex of 1-m-old mice were immunolabeled for lysosome-associated membrane protein 2 (LAMP-2), which is associated with both lysosomes and PLs (33), and NG2 (chondroitin sulfate proteoglycan 4) that specifically labels OPCs (11). Consistent with the ultrastructural observations described above, OPCs at this age contained abundant LAMP-2 immunoreactive circular organelles distributed throughout their somata and processes (mean: 54.2, range min 25, max 94, and n = 14) (*SI Appendix*, Fig. S8 *A* and *B*), indicating a high investment in cellular turnover in the developing cortex.

During the critical period of ocular dominance plasticity in the mouse visual cortex from P21 to P35, microglia engulf and eliminate synapses (34, 35) and remove dead and dying cells through phagocytosis and eventual digestion via acidified phagosomes and PLs (36). This structural remodeling of cortical connections declines rapidly after the first postnatal month (29, 37), coinciding with the end of the critical period. To determine if the incidence of PLs in OPCs also changes over time, we quantified PLs in OPCs reconstructed from serial TEM volumes obtained in the P54 mouse visual cortex. Significantly fewer PLs were present in OPCs at this age (PL density: P36 OPCs, 0.143 ± 0.05 PLs/µm^3^; P54 OPCs, 0.085 ± 0.03 PLs/µm^3^; *P* = 0.01, Student’s *t* test, and n = 9, 11, respectively). Vesicles were also apparent in all P54 OPCs that were examined (mean ± standard deviation, range 13–45%, n = 9 OPCs). Together, these results indicate there is a temporal shift in OPC behavior over time and that OPCs may contribute to an early phase of structural remodeling of neurons during the final phase of the critical period in the visual cortex.

OPCs undergo dramatic changes in gene expression and morphology as they differentiate into myelin forming oligodendrocytes (4). To determine if axonal engulfment is preferentially associated with the OPC progenitor state, we also examined the cytosol of premyelinating and mature oligodendrocytes at P54 (due to the smaller volume, there was only one partially reconstructed premyelinating oligodendrocyte in the P36 dataset). These more mature oligodendroglia were distinguished from OPCs by the lack of primary cilia (23), the presence of significantly more sheets of membrane extending along axons, and membrane wraps indicative of nascent myelination (*SI Appendix*, Fig. S9 *A*–*D*). Analysis of their somata and processes revealed that premyelinating oligodendrocytes had lower densities of PLs than OPCs (*SI Appendix*, Fig. S9 *E* and *F*) (PL density: P54 OPCs, 0.085 ± 0.03 PLs/µm^3^; P54 Premyelinating oligodendrocyte, 0.034 ± 0.018 PLs/µm^3^; *P* = 0.001, Student’s *t* test, n = 11, 5, respectively). In fully mature oligodendrocytes, PLs were extremely rare (*SI Appendix*, Fig. S9 *E* and *F*), suggesting that engulfment of neuronal processes declines rapidly as OPCs undergo differentiation.

## OPCs Express Genes that Enable Phagocytosis

**Fig. 5.**
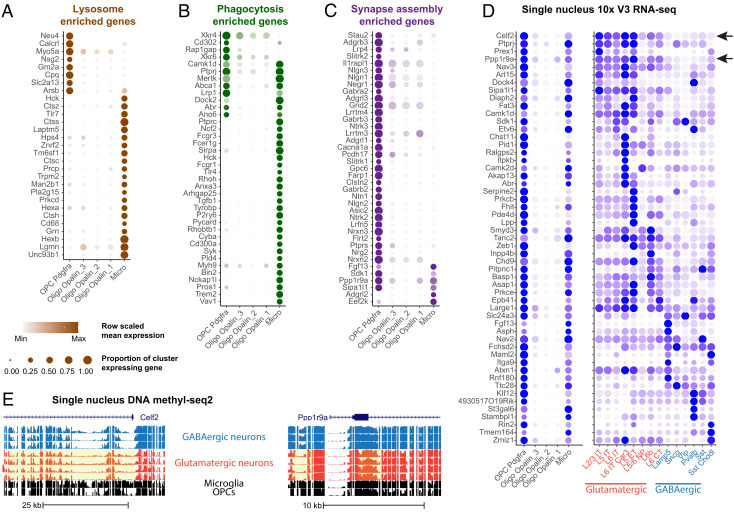
Detection of phagolysosome genes and neuronal transcripts in OPCs in oligodendrocytes and microglia. (*A–C)* Dot plots of enriched lysosome (*A*), phagocytosis (*B*), and synapse assembly (*C*) GO term genes enriched in OPCs and/or microglia, relative to mature oligodendrocytes. (*D*) Dot plot of neuronal subclass marker genes expressed in OPCs, mature oligodendrocytes – Oligo, microglia – Micro, and glutamatergic (red) and GABAergic (blue) neuronal subclasses. (*E*) Genome tracks showing glutamatergic marker genes, *Celf2* (*Top panel*) and *Ppp1r9a* (*Bottom panel*) with hypomethylated chromatin highlighted in yellow. Blue tracks show GABAergic and red tracks show glutamatergic neuronal subclasses.

The 3D EM reconstructions indicate that OPC processes are filled with numerous PLs that contain the neuronal material, suggesting that they assist in remodeling nascent neuronal circuits through engulfment of axons and occasionally synapses. Phagocytosis requires a complex array of proteins to recognize and engulf distinct cargos, the components for which are highly conserved among different species. To further support our EM findings of the presence of PLs in OPCs, we asked whether OPCs express genes that would enable phagocytosis. For this, we analyzed existing single-nucleus RNA-seq and DNA-methylation datasets derived from the mouse motor cortex (P56) (38) for both lysosomal and phagocytic genes (Fig. 5 *A* and *B*). OPC nuclei were identified by coexpression of *pdgfra* and *olig2* and premyelinating/myelinating oligodendrocytes were identified by expression of *opalin* (Fig. 5 *A*–*C*). Expression of genes that encode components of phagocytosis were then compared between OPCs, microglia, and mature oligodendrocytes (Fig. 5 *A* and *B*), identified by previous characterization (38) and expression of distinct marker genes (*SI Appendix*, Fig. S10).

Both OPCs and microglia expressed high levels of mRNAs encoding phagocytic and lysosomal genes. Of the 38 phagocytosis-related genes examined, 32% were expressed in OPCs and 89% were expressed by microglia (Fig. 5*B*). Eight phagocytic genes were expressed in both glial types, including *Mertk*, *Ptprj*, and *Lrp1*. *Mertk* (Mer tyrosine kinase) is a member of the TAM (Tyro3-Axl-Mer) family of receptors that signal engulfment, which astrocytes use to refine connectivity in the adult mouse hippocampus by phagocytosing excitatory synapses (39). *Ptprj* is a tyrosine phosphatase receptor protein that is known to regulate phagocytosis and migration in microglia (40), and *Lrp1* is a low-density lipoprotein receptor essential for myelin phagocytosis and regulation of inflammation in OPCs (41). Several genes that encode phagocytosis-related proteins expressed in OPCs were absent from microglia: *Rap1gap*, a GTPase-activating protein that mediates FcyR-dependent phagocytosis (42); and *Xrkr4* and *Xrkr6*, proteins that promote the exposure of phosphatidylserine to produce “eat me” signaling during phagocytosis, which are highly expressed in the developing brain (43). The distinct complement of phagocytic genes expressed by OPCs and microglia may enable these cells to identify and engulf discrete parts of neurons. Notably, expression of all the phagocytosis-related genes were negligeable in oligodendrocytes, consistent with the much higher incidence of PLs in OPCs. Together the results from electron microscopy, light microscopy, immunohistochemistry staining of cultured OPCs and the transcriptomics results, are consistent in supporting the unexpected finding that OPCs contain phagolysomes and engage in phagocytosis.

## Discussion

Consolidation of nascent neural networks into stable, but adaptable, circuits requires extensive remodeling during development, when excess neurons, axon collaterals, and individual synapses are removed (44–46). Many neuronal connections are made by collateral branches that arise interstitially from axon shafts as filopodia or small sprouts (47). The fine scale removal and refinement of excess branches is essential and is aided by glial cells, which recognize and engulf both cellular debris and portions of intact neurons (48, 49). Unambiguous visualization of engulfment has proven difficult at the light level, because of the small size of membrane-bound organelles like PLs and the complex, tortuous morphology of glial cell processes. Our analysis of new volumetric TEM datasets, in which the thin processes of distinct glial cells could be traced back to their somata to allow cell identification, unexpectedly revealed that OPCs routinely encapsulate axons and contain numerous PLs, suggesting that they engage in axon engulfment during a crucial period in cortical development.

Microglia are considered to be the primary mediators of neuronal structural refinement during development and in the adult CNS. They express phagocytic genes (e.g., *Merktk*, *Dock2*, and *Sirpa*) (50), engage in constant surveillance through process motility, engulf synapses and copious amounts of cellular debris, and accumulate the presynaptic material in their cytoplasm (35, 51). Participation of OPCs in developmental axon ingestion is unexpected, as genetic fate tracing indicates that many, but not all, of these progenitors will eventually die or transform into myelin forming oligodendrocytes (52, 53). It is known that both OPCs and oligodendrocytes are profoundly affected by the activity of axons during development (54) and myelination (55). For example, contact between oligodendrocytes and axons is essential for oligodendrocytes to succeed at myelination and if they fail, they do not survive (54, 56). OPCs persist into adulthood, tiling across the brain and maintaining their ability to migrate and proliferate (4), able to sense injury to existing OLs and respond by division and differentiation (57). While this is often considered their main role in the brain, there is speculation about what other functions they might carry out, since less than1% of OPCs become OLs (24). Nevertheless, OPCs are well positioned to contribute to structural refinement, perhaps by sensing the local environment through their synapses, or by the transfer of axonal material via trogocytosis (58). They share many features with microglia – they are present at a similar density, maintain a grid-like distribution with nonoverlapping domains, possess ramified, radially oriented processes and are highly motile, continuously exploring their surrounding environment with dynamic filopodia (24). Emerging evidence also suggests that adult OPCs do more than serve as progenitors for oligodendrocytes, as they are found in regions where there is no myelin, and like microglia, OPCs migrate to sites of injury and contribute to scar formation (59–61) and present antigen (62). Although several prior studies have reported possible engulfment of cellular debris by OPCs in injury and disease contexts (13, 63), the involvement of these resident progenitors in widespread axon refinement during development has not been reported to our knowledge, but is consistent with another recent studies (64, 65).

This analysis of high-resolution serial EM data from the cerebral cortex shows that OPCs extended fine processes that make numerous contacts with axonal outgrowths and small axon branches primarily of excitatory neurons, surrounding and encircling them. In some morphologically specialized OPCs, neuronal components were enveloped within cup-shaped formations and sheetlike cytoplasmic extensions, reminiscent of noncompact myelin. Furthermore, all OPC processes contained a high density of PLs filled with the material exhibiting characteristic features of synapses, notably 40-nm vesicles, suggesting that OPCs target specific portions of axons for removal. These ingestions could represent trogocytosis, in which small portions of cells are pinched off (66). Although the molecular mechanisms that enable trogocytosis have not been fully defined, OPCs at this age express numerous genes involved in phagocytosis. Together, these data provide evidence that OPCs not only serve as progenitors for oligodendrocytes, but also engage in the remodeling of neuronal processes during a dynamic stage of cortical alteration and maturation. Recent studies suggest that astrocytes also express phagocytic machinery (e.g., *Mertk*, *Megf10*) (67) and participate in excitatory synapse removal in adult mice (68). The nervous system may accelerate circuit refinement by enhancing overall degradative capacity and enable greater spatial and temporal control of this process by recruiting different glial cell types that recognize distinct cellular components.

The robust dynamics and broad distribution of OPCs place them in an ideal position to engage in the modification of neural circuits. Remarkably, OPC processes contained a higher density of PLs than surrounding microglia, raising the possibility that they are responsible for a significant amount of fine scale structural modifications at this stage of maturation of the cortex. However, it is also possible the engulfment and digestion of axonal elements by OPCs proceed more slowly than in microglia, leading to higher phagolysosome accumulation in their branches. It is conceivable that distinct types and temporal mechanisms of phagosome formation, maturation, and progression of lysosomal to phagolysosome formation in the two types of glia may exist but this is not known. This may be reflected in their genomic differences as OPCs express fewer phagocytic and lysosomal receptors. Previous studies documented that OPCs are a diverse and constantly changing population, with continuous cell turnover as a result of differentiation and cell death (24). Could it be that the build of ingested debris in the branches of OPCs leads to programmed cell death since the process of phagocytosis is closely to apoptosis (32)? Since these data represent a snapshot in time and portray only a static position, one can only speculate about mechanisms at work.

Although the presence of PLs within OPC processes declined with age, the persistence of OPCs throughout the CNS, which retain a similar morphology and dynamics, raises the possibility that they may retain the capacity to modify circuit maturation and clear cellular debris induced by injury or normal aging, consistent with conclusions made in other histological studies (69, 70). Some OPCs in this region of the developing cortex will differentiate to form oligodendrocytes that myelinate both excitatory and inhibitory axons (54, 71–73). However, the interactions between neurons and OPCs described here are distinct from the early stages of axon wrapping, as these cells had not yet begun transforming into oligodendrocytes, based on the lack of compacted myelin sheaths and presence of primary cilia, and phagolysosome abundance rapidly declined with differentiation. Whether the interactions described here are an early harbinger of myelination (54, 74), to possibly trim superfluous axon branches prior to axon ensheathment or signal which regions of axons are to be myelinated, are not yet known.

Together, these findings raise new questions about how distinct glial cell types coordinate their phagocytic behavior to shape brain connectivity and contribute to plasticity, homeostasis, and disease processes. The interactions between different glial types and how they might coordinate large- and small-scale pruning activity, is fodder for investigation. Future studies selectively manipulating the ability of OPCs to phagocytose and degrade cellular material at different developmental stages may reveal the complex role of these progenitors in early refinement of neural circuits and lead to a deeper understanding of how the behavior of distinct glial cell types is coordinated to promote the maturation of the nervous system.

### Brief Methods TEM Dataset Production.

Detailed description of methods is available in the supplementary information and also in refs. 7, 15, and 16. All animal procedures were approved by the Institutional Animal Care and Use Committee at the Allen Institute for Brain Science or Baylor College of Medicine.

### TEM Datasets.

We used two densely segmented and reconstructed volumes, obtained from serial section transmission electron microscopy (TEM) in the mouse primary visual cortex. These data sets were collected as part of the IARPA Machine Intelligence from Cortical Network (MICrONS) consortium project (https://www.iarpa.gov/index.php/research-programs/microns) to study the functional connectivity of neurons; however, they also provided rich and unprecedented details of the fine morphology of glial cell types. Here, we use these datasets to evaluate the population of OPCs and microglia in the 3D environment of the mouse brain visual cortex. Dataset 1 was taken from a 200-µm-thick sample containing layer 2/3 of the mouse visual cortex (P36) and measured approximately 250 µm × 140 µm × 90 µm (Fig. 1*A*). Dataset 2 was taken from a 200-μm-thick sample taken from a P54 mouse visual cortex and included all 6 layers, measuring approximately 56 μm × 1 mm × 30 μm. The P36 data set is publicly available at https://microns-explorer.org/. The mice used in this study underwent two-photon imaging as described in *SI Appendix*, *Methods*. There was no evidence of gliosis or ultrastructural damage to sample area caused by either the cortical window or two photon imaging, as previously shown by Holtmatt et al. (75).

#### Mouse line.

Mouse P 36 was a triple-heterozygote for the following three genes: 1) Cre driver: Slc17a7-IRES2_Cre (Jax: 023527<https://www.jax.org/strain/005359>), 2) tTA driver: B6;CBA- Tg(Camk2a-tTA)1Mmay/J (Jax: 003010<https://www.jax.org/strain/003010>), 3) GCaMP6f Reporter: Ai93(JAX 024103) (Allen Institute) (76). Mouse P49 was a cross of: B6;CBA-Tg(Camk2a-tTA)1Mmay/J (Jax: 003010) and B6;DBA-Tg(tetO-GCaMP6s)2Niell/J (Jax: 024742).

#### TEM histology.

The histology protocol used here is based on the work of refs. 77 and 78. The histology protocol used here is based on the work of refs. 77 and 78, with modifications to accommodate different tissue block sizes and to improve tissue contrast for transmission electron microscopy (TEM). This high-contrast protocol avoided the necessity for grid staining. See *SI Appendix*, *Methods* for more details regarding perfusion and sample handling.

#### Ultrathin sectioning and imaging.

The tissue block was trimmed to contain the neurophysiology recording site which is the region of interest (ROI) then sectioned to 40-nm ultrathin sections that were manually collected onto slot grids coated with a 50-nm-thick LUXFilm support or automatically onto a grid tape (79). The imaging platform used for high-throughput serial section imaging was a JEOL-1200EXII 120kV transmission electron microscope that was modified with an extended column, a custom scintillator, and a large format sCMOS camera outfitted with a low distortion lens (80, 81). Subsequent imaging of the scintillator with a high-resolution, large-format camera allowed the capture of fields-of-view as large as 13 × 13 μm at 4-nm resolution.

#### Image volume assembly and morphological segmentation.

Aligning the individual image tiles and sections into a coherent three-dimensional volume and segmenting the cellular morphology dataset was performed as previously described within (5, 16, 82). For further details about the methods, see *SI Appendix*, *Methods*.

#### Axonal analysis.

Using the multicut tool in Neuroglancer, individual branches of ten OPCs were removed at their trunk near the soma. Using the annotation tool in Neuroglancer, phagosomes, PLs, and axon ingestions were tagged along the lengths of fine branchlets. Axon ingestions were still attached to their parent axon, so that the axon could be followed along its length in both 3D and ultrastructural modalities. This made it possible to determine the class of axon, excitatory or inhibitory by examining the ultrastructure of individual boutons. By EM, excitatory axons have a large, electron-dense postsynaptic membrane while inhibitory axon boutons lack this feature, having instead thinner symmetric densities. If there was uncertainty, those were marked as unsure. Since the engulfed axons usually formed multiple synapses, we used the characteristics of the postsynaptic structure as further confirmation of our classification, since excitatory axons make most of their synapses with spines while inhibitory axons make most of their synapses with somata and dendritic shafts. These criteria were also confirmed using the axons of excitatory and inhibitory cells whose soma is present in the volume (see Turner et al., 2022).

## Supplementary Material

Appendix 01 (PDF)

Movie S1 Satellite Glia. All types of glia can be in the satellite position, in close apposition to neuronal cell somas. This movie shows both an OPC and a microglial cell together on the soma of a pyramidal neuron with their glial branches intertwined. https://vimeo.com/747877539

Movie S2 Following an OPC branch through the neuropil. This movie follows the branch of an OPC (Fig.2 *F*) back to its soma. Green ball shows the position in the branch in the insert. Many cellular organelles are present in the branch. https://vimeo.com/747877039

Movie S3 Axon engulfment. The tip of small collateral branch(gray) is engulfed within the cytoplasm of the OPC (pink). The axon tip remains attached the parent axon and that allows its identification as inhibitory. See SI Fig 5 *E–H*. https://vimeo.com/747877154

Movie S4 Spine synapse engulfed by OPC. The dendritic spine in blue makes an excitatory synapse with the axon in gray. Both are still attached to their parent cell. There were few examples of whole synapses being ingested in this dataset. See Fig. 4*G-L*. https://vimeo.com/747877237

## Data Availability

[Electron microscopy images; software and code] data have been deposited in [www.microns-explorer.org; GitHub] (DOI: 10.7554/eLife.73783; All software is open source and available at http://github.com/seung-lab if not otherwise mentioned. Alembic: Stitching and alignment. CloudVolume: Reading and writing volumetric data, meshes, and skeletons to and from the cloud Chunkflow: Running convolutional nets on large datasets DeepEM: Training convolutional nets to detect neuronal boundaries. DynamicAnnotationFramework: Proofreading and connectome updates (visit https://github.com/seung-lab/AnnotationPipelineOverviewforrepositorylist) Igneous: Coordinating downsampling, meshing, and data management. MeshParty: Interaction with meshes and mesh-based skeletonization (https://github.com/sdorkenw/MeshParty) MMAAPP: Watershed, size-dependent single linkage clustering, and mean affinity agglomeration. PyTorchUtils: Training convolutional nets for synapse detection and partner assignment (https://github.com/nicholasturner1/PyTorchUtils) (15). Synaptor: Processing output of the convolutional net for predicting synaptic clefts (https://github.com/nicholasturner1/Synaptor). TinyBrain and zmesh: Downsampling and meshing (precursors of the libraries that were used).).

## References

[1] E. G. Baxi , Lineage tracing reveals dynamic changes in oligodendrocyte precursor cells following cuprizone-induced demyelination. Glia **65**, 2087–2098 (2017). 28940645 10.1002/glia.23229 PMC5761347

[2] D. Bergles, C. E. Jahr, Glutamatergic synapses on oligodendrocyte precursor cells in the hippocampus. Nature **405**, 187–191 (1997). 10.1038/35012083 10821275

[3] S. C. Lin, D. E. Bergles, Synaptic signaling between neurons and glia. Glia **47**, 290–298 (2004). 15252819 10.1002/glia.20060

[4] D. E. Bergles, W. D. Richardson, Oligodendrocyte development and plasticity. Cold Spring Harb. Perspect. Biol. **8**, a020453 (2015). 26492571 10.1101/cshperspect.a020453 PMC4743079

[5] N. L. Turner , Multiscale and multimodal reconstruction of cortical structure and function. bioRxiv [Preprint] (2020). 10.1101/2020.10.14.338681 (Accessed 29 May 2021).

[6] A. Motta , Dense connectomic reconstruction in layer 4 of the somatosensory cortex. Science **366**, 14 (2019). 31649140 10.1126/science.aay3134

[7] N. L. Turner , Reconstruction of neocortex: Organelles, compartments, cells, circuits, and activity. Cell **185**, 1082–1100 (2022). 35216674 10.1016/j.cell.2022.01.023 PMC9337909

[8] S. Mori, C. P. Leblond, Electron microscopic identification of three classes of oligodendrocytes and a preliminary study of their proliferative activity in the corpus callosum of young rats. J. Comp. Neurol. **139**, 1–28 (1970). 4191626 10.1002/cne.901390102

[9] M. R. Dawson, J. M. Levine, R. Reynolds, NG2-expressing cells in the CNS. J. Neurosci. Res. **61**, 471–479 (2000). 10956416 10.1002/1097-4547(20000901)61:5<471::AID-JNR1>3.0.CO;2-N

[10] M. C. Raff, R. H. Miller, M. Noble, A glial progenitor cell that develops in vitro into an astrocyte or an oligodendrocyte depending on culture medium. Nature **303**, 390–396 (1983). 6304520 10.1038/303390a0

[11] W. Y. Ong, J. M. Levine, A light and electron microscopic study of NG2 chondroitin sulfate proteoglycan-positive oligodendrocyte precursor cells in the normal and kainate-lesioned rat hippocampus. Neuroscience **92**, 83–95 (1999). 10392832 10.1016/s0306-4522(98)00751-9

[12] A. Peters, A fourth type of neurogial cell in the adult central nervous system. J. Neurocytol. **33**, 345–357 (2004). 15475689 10.1023/B:NEUR.0000044195.64009.27

[13] J. E. Vaughn, P. L. Hinds, R. P. Skoff, Electron microscopic studies of Wallerian degeneration in rat optic nerves. I. The multipotential glia. J. Comp. Neurol. **140**, 175–206 (1970). 4319510 10.1002/cne.901400204

[14] A. M. Butt , Cells expressing the NG2 antigen contact nodes of Ranvier in adult CNS white matter. Glia **26**, 84–91 (1999). 10088675

[15] C. M. Schneider-Mizell , Structure and function of axo-axonic inhibition. Elife **185**, 1082–1100 (2021). 10.7554/eLife.73783 PMC8758143 34851292

[16] S. Dorkenwald, Binary and analog variation of synapses between cortical pyramidal neurons. bioRxiv [Preprint] (2019) 10.1101/2019.12.29.890319 (Accessed 15 August 2022). PMC9704804 36382887

[17] E. A. Bushong, M. E. Martone, Y. Z. Jones, M. H. Ellisman, Protoplasmic astrocytes in CA1 stratum radiatum occupy separate anatomical domains. J. Neurosci. **22**, 183–192 (2002). 11756501 10.1523/JNEUROSCI.22-01-00183.2002 PMC6757596

[18] A. Nimmerjahn, F. Kirchhoff, F. Helmchen, Resting microglial cells are highly dynamic surveillants of brain parenchyma in vivo. Science **308**, 1314–1318 (2005). 15831717 10.1126/science.1110647

[19] R. Ventura, K. M. Harris, Three-dimensional relationships between hippocampal synapses and astrocytes. J. Neurosci. **19**, 6897–6906 (1999). 10436047 10.1523/JNEUROSCI.19-16-06897.1999 PMC6782870

[20] D. P. Schafer, E. K. Lehrman, B. Stevens, The "quad-partite" synapse: Microglia-synapse interactions in the developing and mature CNS. Glia **61**, 24–36 (2013). 22829357 10.1002/glia.22389 PMC4082974

[21] A. Battefeld, J. Klooster, M. H. Kole, Myelinating satellite oligodendrocytes are integrated in a glial syncytium constraining neuronal high-frequency activity. Nat. Commun. **7**, 11298 (2016). 27161034 10.1038/ncomms11298 PMC4866043

[22] E. Wogram , Satellite microglia show spontaneous electrical activity that is uncorrelated with activity of the attached neuron. Eur. J. Neurosci. **43**, 1523–1534 (2016). 27060918 10.1111/ejn.13256

[23] P. Falcon-Urrutia, C. M. Carrasco, P. Lois, V. Palma, A. D. Roth, Shh signaling through the primary cilium modulates rat oligodendrocyte differentiation. PLoS One **10**, e0133567 (2015). 26218245 10.1371/journal.pone.0133567 PMC4517900

[24] E. G. Hughes, S. H. Kang, M. Fukaya, D. E. Bergles, Oligodendrocyte progenitors balance growth with self-repulsion to achieve homeostasis in the adult brain. Nat. Neurosci. **16**, 668–676 (2013). 23624515 10.1038/nn.3390 PMC3807738

[25] J. M. Kinchen, K. S. Ravichandran, Phagosome maturation: going through the acid test. Nat. Rev. Mol. Cell Biol. **9**, 781–795 (2008). 18813294 10.1038/nrm2515 PMC2908392

[26] R. Levin, S. Grinstein, J. Canton, The life cycle of phagosomes: formation, maturation, and resolution. Immunol. Rev. **273**, 156–179 (2016). 27558334 10.1111/imr.12439

[27] M. Simons, K. A. Nave, Oligodendrocytes: Myelination and axonal support. Cold Spring Harb. Perspect. Biol. **8**, a020479 (2015). 26101081 10.1101/cshperspect.a020479 PMC4691794

[28] N. Snaidero, M. Simons, Myelination at a glance. J. Cell Sci. **127**, 2999–3004 (2014). 25024457 10.1242/jcs.151043

[29] D. P. Schafer , Microglia sculpt postnatal neural circuits in an activity and complement-dependent manner. Neuron **74**, 691–705 (2012). 22632727 10.1016/j.neuron.2012.03.026 PMC3528177

[30] M. E. Tremblay, R. L. Lowery, A. K. Majewska, Microglial interactions with synapses are modulated by visual experience. PLoS Biol. **8**, e1000527 (2010). 21072242 10.1371/journal.pbio.1000527 PMC2970556

[31] A. N. Hughes, B. Appel, Microglia phagocytose myelin sheaths to modify developmental myelination. Nat. Neurosci. **23**, 1055–1066 (2020). 32632287 10.1038/s41593-020-0654-2 PMC7483351

[32] M. Marquez-Ropero, E. Benito, A. Plaza-Zabala, A. Sierra, Microglial corpse clearance: Lessons from macrophages. Front. Immunol. **11**, 506 (2020). 32292406 10.3389/fimmu.2020.00506 PMC7135884

[33] K. K. Huynh , LAMP proteins are required for fusion of lysosomes with phagosomes. EMBO J. **26**, 313–324 (2007). 17245426 10.1038/sj.emboj.7601511 PMC1783450

[34] G. O. Sipe , Microglial P2Y12 is necessary for synaptic plasticity in mouse visual cortex. Nat. Commun. **7**, 10905 (2016). 26948129 10.1038/ncomms10905 PMC4786684

[35] R. C. Paolicelli , Synaptic pruning by microglia is necessary for normal brain development. Science **333**, 1456–1458 (2011). 21778362 10.1126/science.1202529

[36] F. Peri, C. Nusslein-Volhard, Live imaging of neuronal degradation by microglia reveals a role for v0-ATPase a1 in phagosomal fusion in vivo. Cell **133**, 916–927 (2008). 18510934 10.1016/j.cell.2008.04.037

[37] J. S. Espinosa, M. P. Stryker, Development and plasticity of the primary visual cortex. Neuron **75**, 230–249 (2012). 22841309 10.1016/j.neuron.2012.06.009 PMC3612584

[38] Z. Yao , A taxonomy of transcriptomic cell types across the isocortex and hippocampal formation. Cell **184**, 3222–3241.e3226 (2021). 34004146 10.1016/j.cell.2021.04.021 PMC8195859

[39] A. Mo , Epigenomic signatures of neuronal diversity in the mammalian brain. Neuron **86**, 1369–1384 (2015). 26087164 10.1016/j.neuron.2015.05.018 PMC4499463

[40] N. Schneble , The protein-tyrosine phosphatase DEP-1 promotes migration and phagocytic activity of microglial cells in part through negative regulation of fyn tyrosine kinase. Glia **65**, 416–428 (2017). 27859601 10.1002/glia.23100

[41] A. Fernandez-Castaneda , The active contribution of OPCs to neuroinflammation is mediated by LRP1. Acta. Neuropathol. **139**, 365–382 (2020). 31552482 10.1007/s00401-019-02073-1 PMC6994364

[42] C. J. Fitzer-Attas , Fcgamma receptor-mediated phagocytosis in macrophages lacking the Src family tyrosine kinases Hck, Fgr, and Lyn. J. Exp. Med. **191**, 669–682 (2000). 10684859 10.1084/jem.191.4.669 PMC2195832

[43] J. Suzuki, E. Imanishi, S. Nagata, Exposure of phosphatidylserine by Xk-related protein family members during apoptosis. J. Biol. Chem. **289**, 30257–30267 (2014). 25231987 10.1074/jbc.M114.583419 PMC4215210

[44] Y. Pan, M. Monje, Activity shapes neural circuit form and function: A historical perspective. J. Neurosci. **40**, 944–954 (2020). 31996470 10.1523/JNEUROSCI.0740-19.2019 PMC6988998

[45] J. A. Stogsdill, C. Eroglu, The interplay between neurons and glia in synapse development and plasticity. Curr. Opin. Neurobiol. **42**, 1–8 (2017). 27788368 10.1016/j.conb.2016.09.016 PMC5316301

[46] X. Wu , GABA signaling promotes synapse elimination and axon pruning in developing cortical inhibitory interneurons. J. Neurosci. **32**, 331–343 (2012). 22219294 10.1523/JNEUROSCI.3189-11.2012 PMC3742883

[47] L. Luo, D. D. O’Leary, Axon retraction and degeneration in development and disease. Annu. Rev. Neurosci. **28**, 127–156 (2005). 16022592 10.1146/annurev.neuro.28.061604.135632

[48] S. Raiders , Engulfed by glia: Glial pruning in development, function, and injury across species. J. Neurosci. **41**, 823–833 (2021). 33468571 10.1523/JNEUROSCI.1660-20.2020 PMC7880271

[49] D. L. Bishop, T. Misgeld, M. K. Walsh, W. B. Gan, J. W. Lichtman, Axon branch removal at developing synapses by axosome shedding. Neuron **44**, 651–661 (2004). 15541313 10.1016/j.neuron.2004.10.026

[50] A. Sierra, O. Abiega, A. Shahraz, H. Neumann, Janus-faced microglia: Beneficial and detrimental consequences of microglial phagocytosis. Front. Cell Neurosci. **7**, 6 (2013). 23386811 10.3389/fncel.2013.00006 PMC3558702

[51] D. K. Wilton, L. Dissing-Olesen, B. Stevens, Neuron-glia signaling in synapse elimination. Annu. Rev. Neurosci. **42**, 107–127 (2019). 31283900 10.1146/annurev-neuro-070918-050306

[52] S. H. Kang, M. Fukaya, J. K. Yang, J. D. Rothstein, D. E. Bergles, NG2+ CNS glial progenitors remain committed to the oligodendrocyte lineage in postnatal life and following neurodegeneration. Neuron **68**, 668–681 (2010). 21092857 10.1016/j.neuron.2010.09.009 PMC2989827

[53] B. A. Barres , Cell death and control of cell survival in the oligodendrocyte lineage. Cell **70**, 31–46 (1992). 1623522 10.1016/0092-8674(92)90531-g

[54] B. A. Barres, M. C. Raff, Axonal control of oligodendrocyte development. J. Cell Biol. **147**, 1123–1128 (1999). 10601327 10.1083/jcb.147.6.1123 PMC2168096

[55] D. A. Lyons, W. S. Talbot, Glial cell development and function in zebrafish. Cold Spring Harb. Perspect. Biol. **7**, a020586 (2014). 25395296 10.1101/cshperspect.a020586 PMC4315925

[56] E. G. Hughes, M. E. Stockton, Premyelinating oligodendrocytes: Mechanisms underlying cell survival and integration. Front. Cell Dev. Biol. **9**, 714169 (2021). 34368163 10.3389/fcell.2021.714169 PMC8335399

[57] K. L. Adams, V. Gallo, The diversity and disparity of the glial scar. Nat. Neurosci. **21**, 9–15 (2018). 29269757 10.1038/s41593-017-0033-9 PMC5937232

[58] E. Uribe-Querol, C. Rosales, The multiple roles of trogocytosis in immunity, the nervous system, and development. Biomed. Res. Int. **2021**, 1601565 (2021). 34604381 10.1155/2021/1601565 PMC8483919

[59] A. M. Tan, W. Zhang, J. M. Levine, NG2: A component of the glial scar that inhibits axon growth. J. Anat. **207**, 717–725 (2005). 16367799 10.1111/j.1469-7580.2005.00452.x PMC1571583

[60] Z. C. Hesp , Proliferating NG2-cell-dependent angiogenesis and scar formation alter axon growth and functional recovery after spinal cord injury in mice. J. Neurosci. **38**, 1366–1382 (2018). 29279310 10.1523/JNEUROSCI.3953-16.2017 PMC5815343

[61] X. Jin, T. R. Riew, H. L. Kim, J. H. Choi, M. Y. Lee, Morphological characterization of NG2 glia and their association with neuroglial cells in the 3-nitropropionic acid-lesioned striatum of rat. Sci. Rep. **8**, 5942 (2018). 29654253 10.1038/s41598-018-24385-0 PMC5899159

[62] L. Kirby , Oligodendrocyte precursor cells present antigen and are cytotoxic targets in inflammatory demyelination. Nat. Commun. **10**, 3887 (2019). 31467299 10.1038/s41467-019-11638-3 PMC6715717

[63] J. W. Fawcett, R. A. Asher, The glial scar and central nervous system repair. Brain Res. Bulletin **49**, 377–391 (1999). 10.1016/s0361-9230(99)00072-6 10483914

[64] Y. S. S. Auguste, Oligodendrocyte precursor cells engulf synaptic inputs in an experience- and microglia dependent manner. bioRxiv [Preprint] (2022). 10.1101/2022.02.10.479887 (Accessed 17 February 2022)

[65] Y. Xiao, L. Petrucco, L. J. Hoodless, R. Portugues, T. Czopka, Oligodendrocyte precursor cells sculpt the visual system by regulating axonal remodeling. Nat. Neurosci. **25**, 280–284 (2022). 35241802 10.1038/s41593-022-01023-7 PMC8904260

[66] T. K. Lim, E. S. Ruthazer, Microglial trogocytosis and the complement system regulate axonal pruning in vivo. Elife **10**, e62167 (2021). 33724186 10.7554/eLife.62167 PMC7963485

[67] W. S. Chung , Astrocytes mediate synapse elimination through MEGF10 and MERTK pathways. Nature **504**, 394–400 (2013). 24270812 10.1038/nature12776 PMC3969024

[68] J. H. Lee , Astrocytes phagocytose adult hippocampal synapses for circuit homeostasis. Nature **590,** 612–617 (2020), 10.1038/s41586-020-03060-3 33361813 10.1038/s41586-020-03060-3

[69] A. M. Falcao , Disease-specific oligodendrocyte lineage cells arise in multiple sclerosis. Nat. Med. **24**, 1837–1844 (2018). 30420755 10.1038/s41591-018-0236-y PMC6544508

[70] E. G. Hughes, J. L. Orthmann-Murphy, A. J. Langseth, D. E. Bergles, Myelin remodeling through experience-dependent oligodendrogenesis in the adult somatosensory cortex. Nat. Neurosci. **21**, 696–706 (2018). 29556025 10.1038/s41593-018-0121-5 PMC5920726

[71] K. D. Micheva , A large fraction of neocortical myelin ensheathes axons of local inhibitory neurons. Elife **5**, e15784 (2016). 27383052 10.7554/eLife.15784 PMC4972537

[72] G. S. Tomassy , Distinct profiles of myelin distribution along single axons of pyramidal neurons in the neocortex. Science **344**, 319–324 (2014). 24744380 10.1126/science.1249766 PMC4122120

[73] S. Timmler, M. Simons, Grey matter myelination. Glia **67**, 2063–2070 (2019). 30860619 10.1002/glia.23614

[74] H. Wake , Nonsynaptic junctions on myelinating glia promote preferential myelination of electrically active axons. Nat. Commun. **6**, 7844 (2015). 26238238 10.1038/ncomms8844 PMC4532789

[75] A. Holtmaat , Long-term, high-resolution imaging in the mouse neocortex through a chronic cranial window. Nat. Protoc. **4**, 1128–1144 (2009). 19617885 10.1038/nprot.2009.89 PMC3072839

[76] L. Madisen , Transgenic mice for intersectional targeting of neural sensors and effectors with high specificity and performance. Neuron **85**, 942–958 (2015). 25741722 10.1016/j.neuron.2015.02.022 PMC4365051

[77] Y. Hua, P. Laserstein, M. Helmstaedter, Large-volume en-bloc staining for electron microscopy-based connectomics. Nat. Commun. **6**, 7923 (2015). 26235643 10.1038/ncomms8923 PMC4532871

[78] J. C. Tapia , High-contrast en bloc staining of neuronal tissue for field emission scanning electron microscopy. Nat. Protoc. **7**, 193–206 (2012). 22240582 10.1038/nprot.2011.439 PMC3701260

[79] J. S. Phelps , Reconstruction of motor control circuits in adult Drosophila using automated transmission electron microscopy. Cell **184**, 759–774.e718 (2021). 33400916 10.1016/j.cell.2020.12.013 PMC8312698

[80] D. D. Bock , Network anatomy and in vivo physiology of visual cortical neurons. Nature **471**, 177–182 (2011). 21390124 10.1038/nature09802 PMC3095821

[81] W. Yin , A petascale automated imaging pipeline for mapping neuronal circuits with high-throughput transmission electron microscopy. Nat. Commun. **11**, 4949 (2020). 33009388 10.1038/s41467-020-18659-3 PMC7532165

[82] C. M. Schneider-Mizell, Chandelier cell anatomy and function reveal a variably distributed but common signal. bioRxiv [Preprint] (2020). 10.1101/2020.03.31.018952 (Accessed 10 October 2022)

