# Supplementary material for: Oligodendrocyte precursor cells ingest axons in the mouse neocortex
Source: Proc Natl Acad Sci U S A. 2022 Nov 23;119(48):e2202580119. doi: 10.1073/pnas.2202580119 (PMC9889886; doi:10.1073/pnas.2202580119)
Supplement: Supplementary file 1 — Appendix 01 (PDF) [file pnas.2202580119.sapp.pdf]

PNAS

Supporting Information for

Oligodendrocyte precursor cells ingest axons in the mouse neocortex

JoAnn Buchanan[1,7*], Leila Elabbady[1], Forrest Collman[1], Nikolas L. Jorstad[1] ,Trygve E. Bakken[1], Carolyn Ott[2], Jenna Glatzer[3], Adam A. Bleckert[1], Agnes L. Bodor[1], Derrick Brittain[1], Daniel J. Bumbarger[1], Gayathri Mahalingam[1], Sharmishtaa Seshamani[1], Casey Schneider-Mizell[1], Marc M. Takeno [1], Russel Torres[1], Wenjing Yin[1], Rebecca D. Hodge[1], Manuel Castro[4], Sven Dorkenwald[4,5], Dodam Ih[4], Chris S. Jordan[4], Nico Kemnitz[4], Kisuk Lee[4,5], Ran Lu[4], Thomas Macrina[4,5], Shang Mu[4], Sergiy Popovych[5], William M. Silversmith[5], Ignacio Tartavull[5], Nicholas L. Turner[4,5] Alyssa M. Wilson[4], William Wong[4], Jingpeng Wu[4], Aleksandar Zlateski[4], Jonathan Zung[4], Jennifer Lippincott-Schwartz[2], Ed S.Lein[1], H. Sebastian Seung[4,5] Dwight E. Bergles[3, 6], R. Clay Reid[1], Nuno Maçarico da Costa[1,7*]

Corresponding author : JoAnn Buchanan, Allen Institute joannb@alleninstitute.org

This PDF file includes:

Figures S1 to S10
Table S1
Legends for Movies S1 to S4

Other supporting materials for this manuscript include the following:

Links to Movies S1 to S4

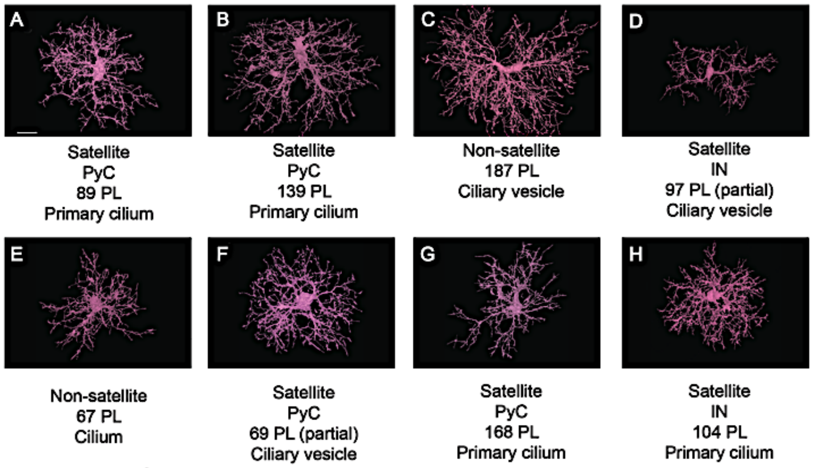

I

Cell	Position	Main branch #	Endings	PL #	PL Vesicle #	Primary cilium
1	Satellite	12	Filopodia	89	47	Yes
2	Satellite	15	Filopodia	139	45	Yes
3	Non-Satellite	15	Cups, wraps	187	44	Ciliary vesicle
4	Satellite	13 partial	Filopodia	97	43	Ciliary vesicle
5	Non-Satellite associated w PyC	11 partial	Filopodia	67	22	Yes
6	Satellite	13	Cups, wraps	69	15	Ciliary vesicle
7	Satellite	17	Filopodia	47	25	Yes
8	Satellite	11	Filopodia	168	45	Yes, submerged
9	Satellite	17	Filopodia	104	60	Yes

SI Fig. 1.
Phagolysosome counts and OPC features. (*A-H*). Vignettes of 8 of 16 OPCs used for analysis in the P36 dataset**.** The ninth cell is pictured in Fig. 2*A***.** Scale bar for all cells, 15 µm. **(***I***)** Cells were scored for satellite position, types of endings, number of main branches, number of PLs, number of PLs containing vesicles and presence of primary cilia.

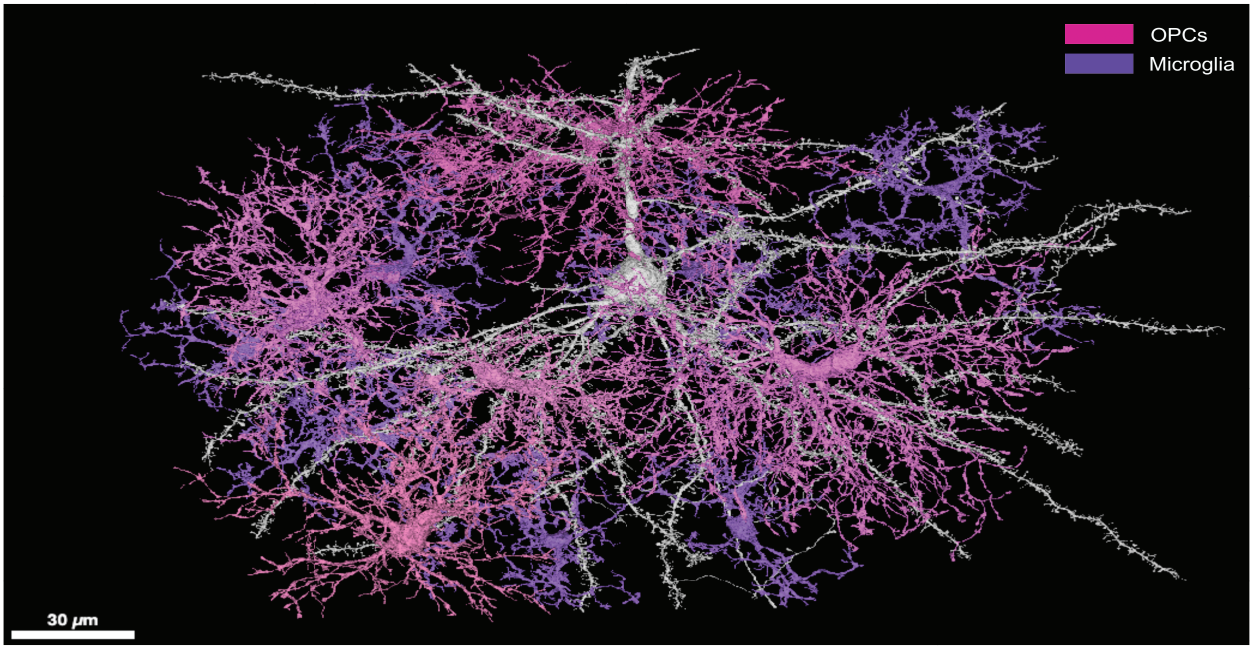

SI Fig 2.
Microglia and OPCs tile across the brain. OPCs(pink) and microglia(purple) occupy distinct sites, with the microglia avoiding other microglia as do OPCs. But they invade each other's territories, with microglia and OPCs intertwining each other's branches. Pyramidal neuron in gray. Scale bar, 30 µm.

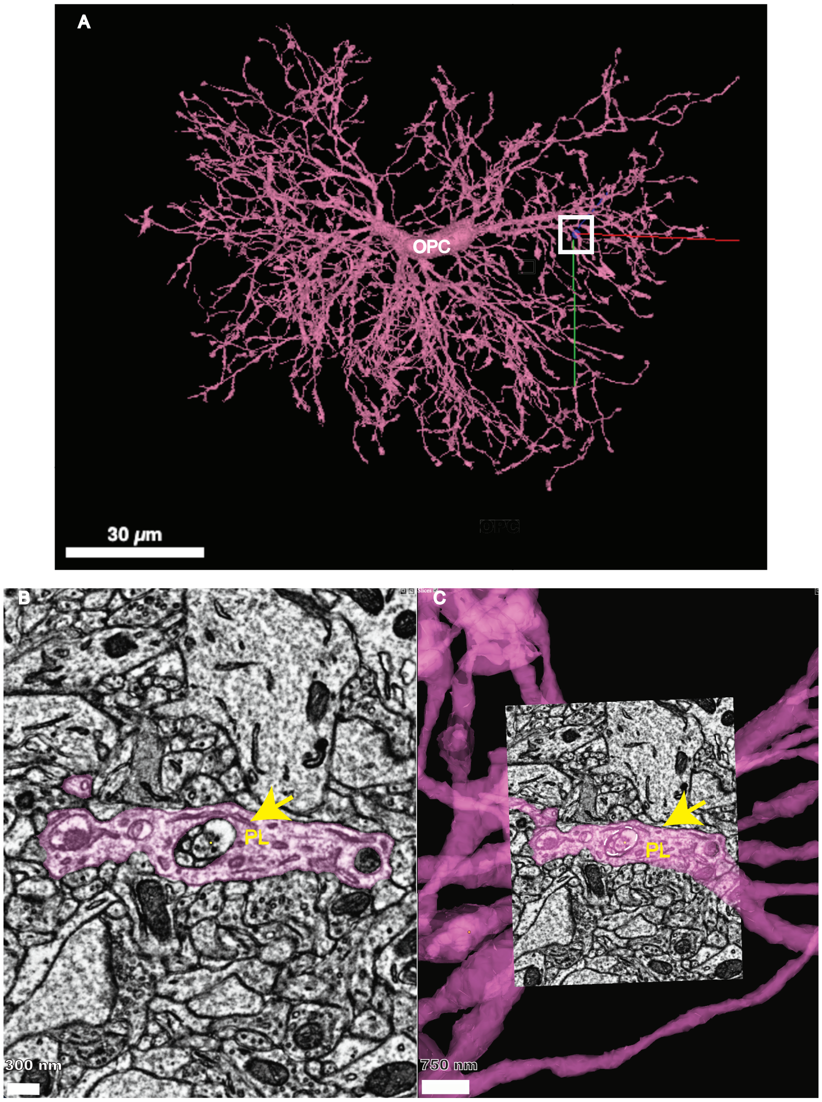

SI Fig. 3.
OPC branches contain phagolysosomes.(*A)*The boxed area of the OPC pictured in Fig. 2F marks the area of the phagolysosome (PL) in the serial sections shown in Fig. 2 D. (*B*) A segment of the branch containing that same PL (yellow arrow)shown in serial sections in Fig. 2 D. Scale bar 300 nm. (*C*) That same PL (yellow arrow) within the OPC branch in pink shown again in the 3 D rendering. Scale bar 700 nm. See SI movie 2.

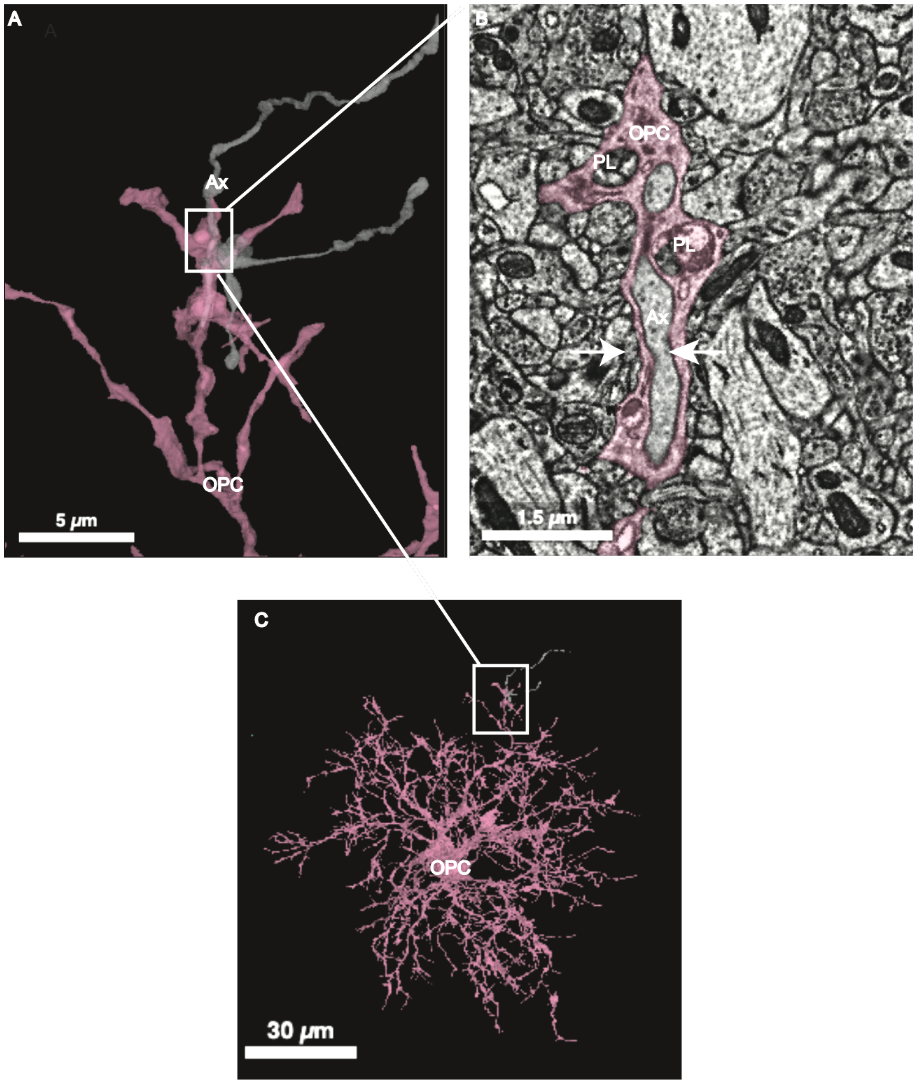

SI. Fig. 4.
An OPC branch ingests a collateral branch as shown in Fig.3. (*A*) In zoomed out view in 3 D the inhibitory axon (Ax) in gray is encased within OPC cytoplasm in pink. Scale bar 5 µm. (*B*) The thin slice view of the same area in (*A*). White arrows point to ingested axon (Ax) in gray. Phagolysosomes (PL) are nearby. Scale bar 1.5 µm. (*C*) Whole OPC cell with the boxed area the same as in (*A*) and (*B*) and in Fig.3. Scale bar 30 µm.

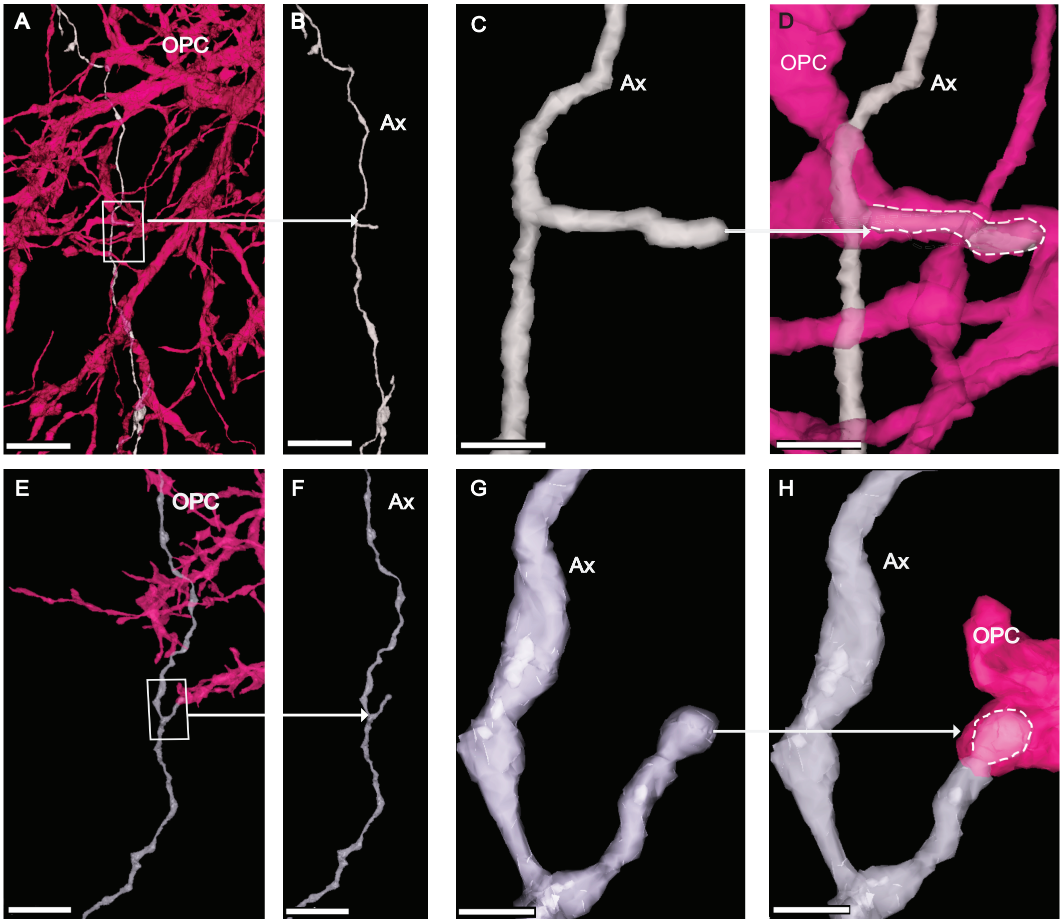

SI Fig. 5.
OPCs ingest collateral branches. **(***A***)** OPC (pink) contacts an excitatory axon (gray). Boxed area shows site of ingestion. Scale bar, 5 µm. (*B*) 3 D rendering of the excitatory axon (gray). Arrow points to collateral branch in boxed area in (*A*). Scale bar, 5 µm. (*C*) Higher magnification of collateral branch. White arrow points to ingested branch in (D). Scale bar, 750 nm. (*D*) 3D rendering of excitatory axon (gray) ingested within the branch of an OPC (pink). Dotted line indicates the outline of ingested collateral branch. Scale bar, 750 nm. (*E*) OPC (pink) contacts inhibitory axon (gray). Boxed area shows site of ingestion. Scale bar, 5 µm. (*F*) 3D rendering of inhibitory axon(Ax) (gray). Arrow points to collateral branch in boxed area in (E). Scale bar, 5 µm. (*G)* Higher magnification of collateral branch. White arrow points to ingested branch in (*E*). Scale bar, 750 nm. (*H*) 3D rendering of inhibitory axon (gray) ingested within the branch of an OPC (pink). Dotted line indicates the outline of ingested collateral branch. Scale bar, 750 nm. See SI movie 3.

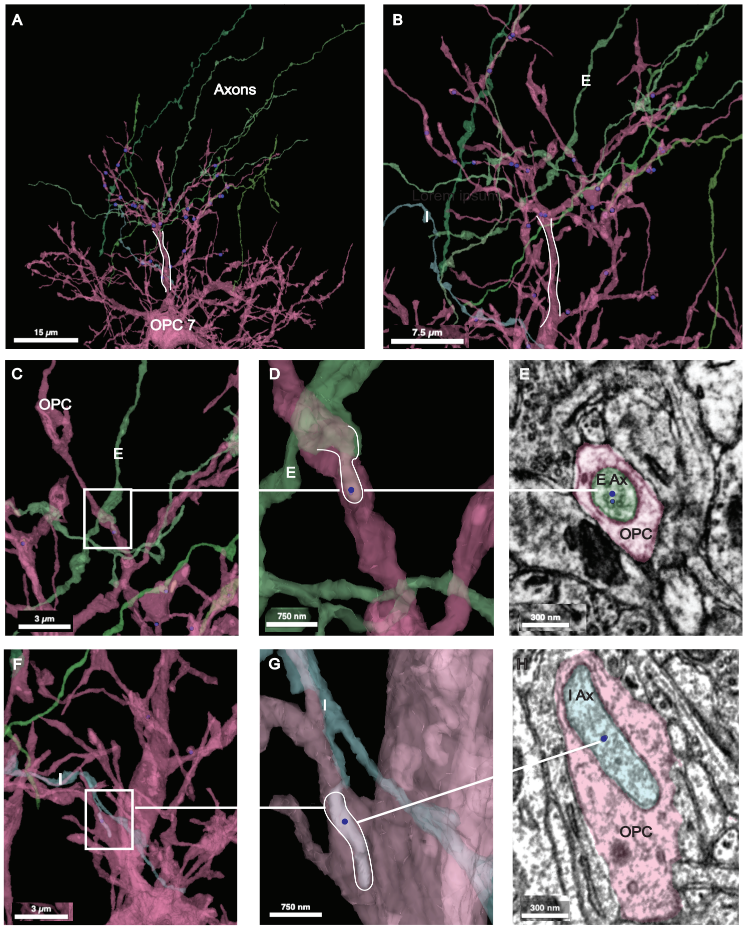

SI Fig. 6.

Axons are engulfed within OPC main branch. **(***A***)** One branch (white outline marks trunk) of OPC 7 (pink) was used to annotate axons engulfed within OPC cytoplasm. Scale bar, 15 µm. (*B*) Higher magnification view of OPC branch trunk (white outline) and its processes (pink) and excitatory axons(E) in green. Scale bar, 7.5 µm**. (***C***)** A portion of an excitatory axon (boxed area) is engulfed within the OPC cytoplasm(pink). Scale bar, 3 µm. (*D*) Higher magnification view of boxed area shows engulfed tip (white outline, blue dot) of excitatory axon (E). Scale bar, 750 nm. **(***E***)** Ultrathin section view of cross section of the green excitatory axon (E Ax) inside the OPC process (white line, blue dot). Scale bar, 300 nm. (*F***)** A portion of an inhibitory (I) axon in blue (boxed area) is engulfed within OPC cytoplasm(pink). Scale bar, 3 µm. (*G*) Higher magnification view of boxed area shows portion of the engulfed inhibitory axon branch (white outline, blue dot) within the OPC cytoplasm(pink). Scale bar, 750 nm. (*H*) Ultrathin section view shows the inhibitory axon (I Ax) (white line, blue dot) surrounded by OPC cytoplasm(pink). Scale bar, 300 nm.

Number and size of axonal ingestions by OPCs

Cell Number	300 nm	500 nm	1 µm	2 µm	< 2 µm
1	11	10	7	2	3
2	9	9	2	3	4
3	1	4	0	0	0
4	6	7	1	1	0
5	4	5	2	4	1
6	0	3	0	0	0
7	2	4	0	1	0
8	13	2	1	1	1
9	7	9	6	0	2
10	7	5	0	0	2

SI Table 1.
The size and numbers of 165 axonal ingestions by ten OPC isolated individual branches. Total number of individual axons examined was 195 and the majority of the ingestion sizes were less than 1µm.

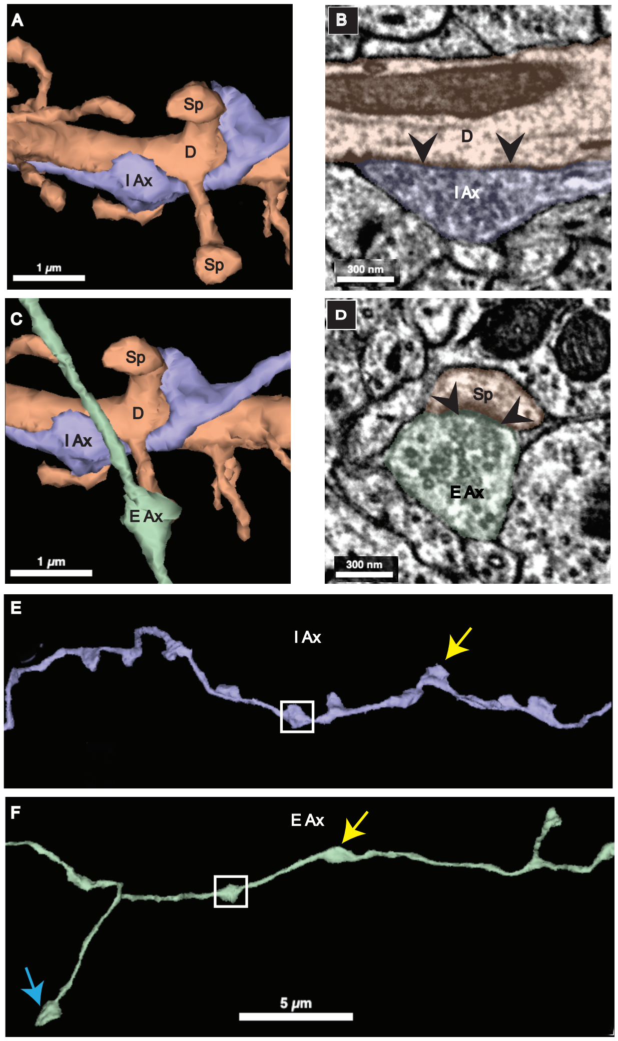

SI Fig. 7.
Morphology of inhibitory and excitatory axons.
(*A*)3D rendering of an inhibitory axon (IAx) in purple. The purple inhibitory axon makes a synapse with the dendritic shaft (orange). Two dendritic spines (Sp) are evident. Scale bar 1µm. (*B*) Ultrathin section view of same area in (*A*) shows synaptic connection between inhibitory axon (IAx) in purple and the dendritic trunk(D) in orange. Note the thin post synaptic density in the dendritic membrane. Scale bar 300 nm. (*C)* An excitatory axon (EAx) in green contacts a synaptic spine (Sp) (orange). Scale bar 1µm. (*D*)Ultrathin section view shows synaptic connection between excitatory axon in green and orange dendritic spine (Sp). Note post synaptic density on the spine head (arrowheads). Scale bar, 300 nm. (*E*) 3D rendering of the inhibitory axon (IAx) in purple shows *en passant* axonal bouton (yellow arrow). The area in panels *A,B* is boxed. (*F*) 3D rendering of an excitatory axon (EAx) shows *en passant* (yellow arrow) and terminal boutons (blue arrow). Boxed area shows area in *C,D*. Scale bars in *C, D*, 5µm.

A

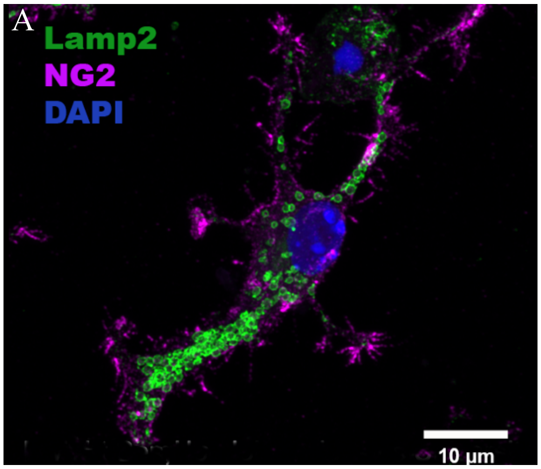

B

File Name	LAMP2+ Structures in Soma #	LAMP2+ Structures in Processes #	Total Number
2	18	7	25
4	48	2	5
8	34	13	47
9	29	28	57
1	6	27	33
2	21	14	35
3	21	21	42
4	23	53	76
5	34	60	94
6	14	28	42
7	13	18	31
8	34	20	54
9	37	11	48
12	20	64	84

SI Fig. 8.
Immunostaining of lysosomes and phagolysosomes in OPCs. (*A*) Primary cultured OPC, immunolabeled with Lamp2 (green) and NG2 chondroitin-sulfate proteoglycan (fuchsia), nucleus stained in blue (DAPI). Scale bar 10 µm. (*B*) Quantification of Lamp2 positive organelles (lysosomes and phagolysosomes) in OPC soma and branches.

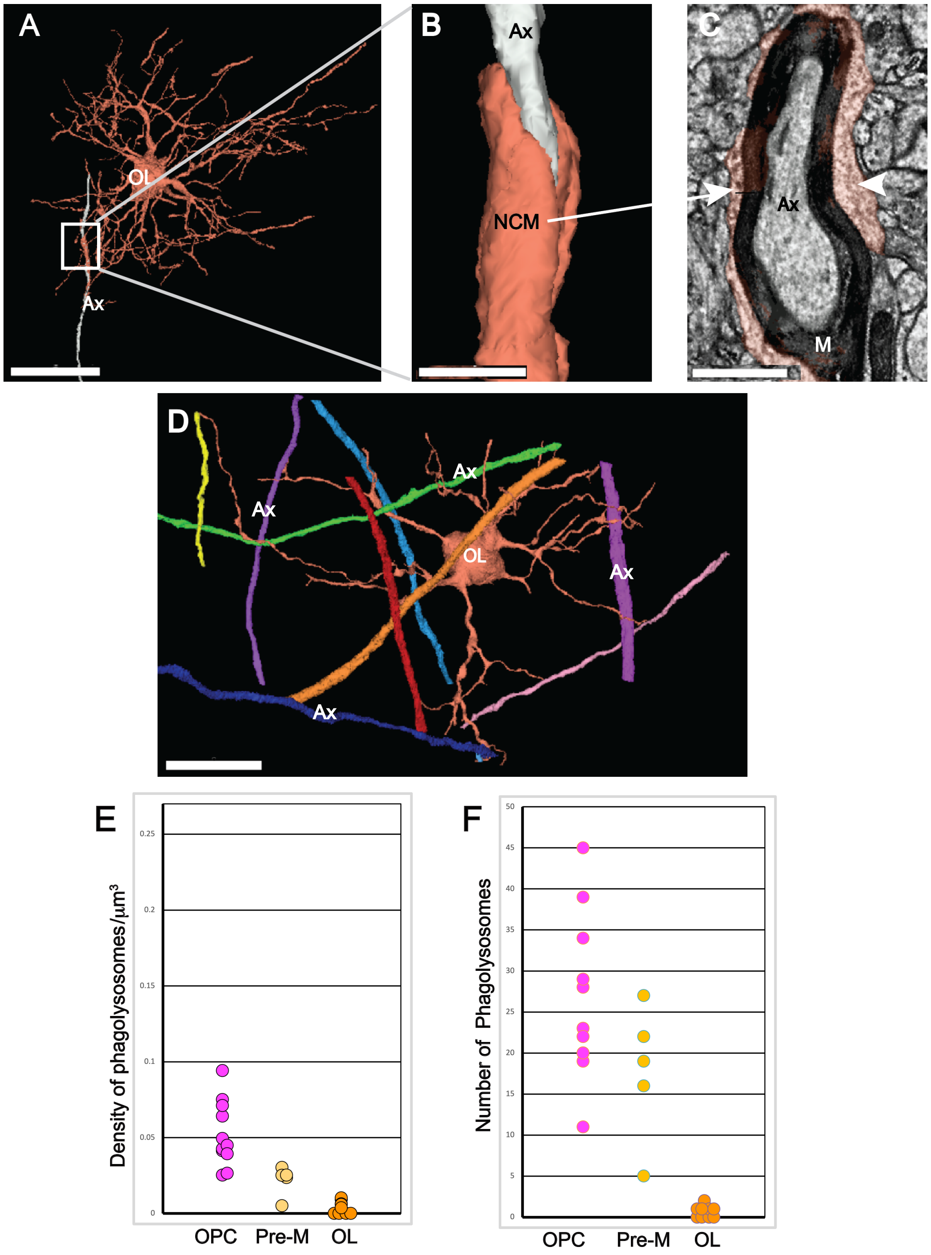

SI Fig. 9
Premyelinating cells, myelinating oligodendrocytes, and phagolysosome densities. **(***A***)** 3D rendering of a premyelinating oligodendrocyte (OL) in P54 dataset shows a branch aligned with an axon (white) (boxed area). Scale bar, 20 µm. (*B*) Higher magnification view of boxed area shows noncompacted myelin (NCM) (orange) and axon (white). Scale bar, 1.5 µm. (*C***)** Ultrathin section view shows the noncompacted myelin(orange)(arrows) on the outside of the compact myelin (black). Scale bar, 750 nm. (*D*) Mature myelinating oligodendrocyte (OL) has thin branches that are aligned with colored myelinated axons (Ax). Scale bar, 10µm. (*E-F*) Plots comparing number and densities of phagolysosome distribution in OPCs (pink dots), premyelinating cells (gold dots) and mature oligodendrocytes (orange dots) P54 dataset.

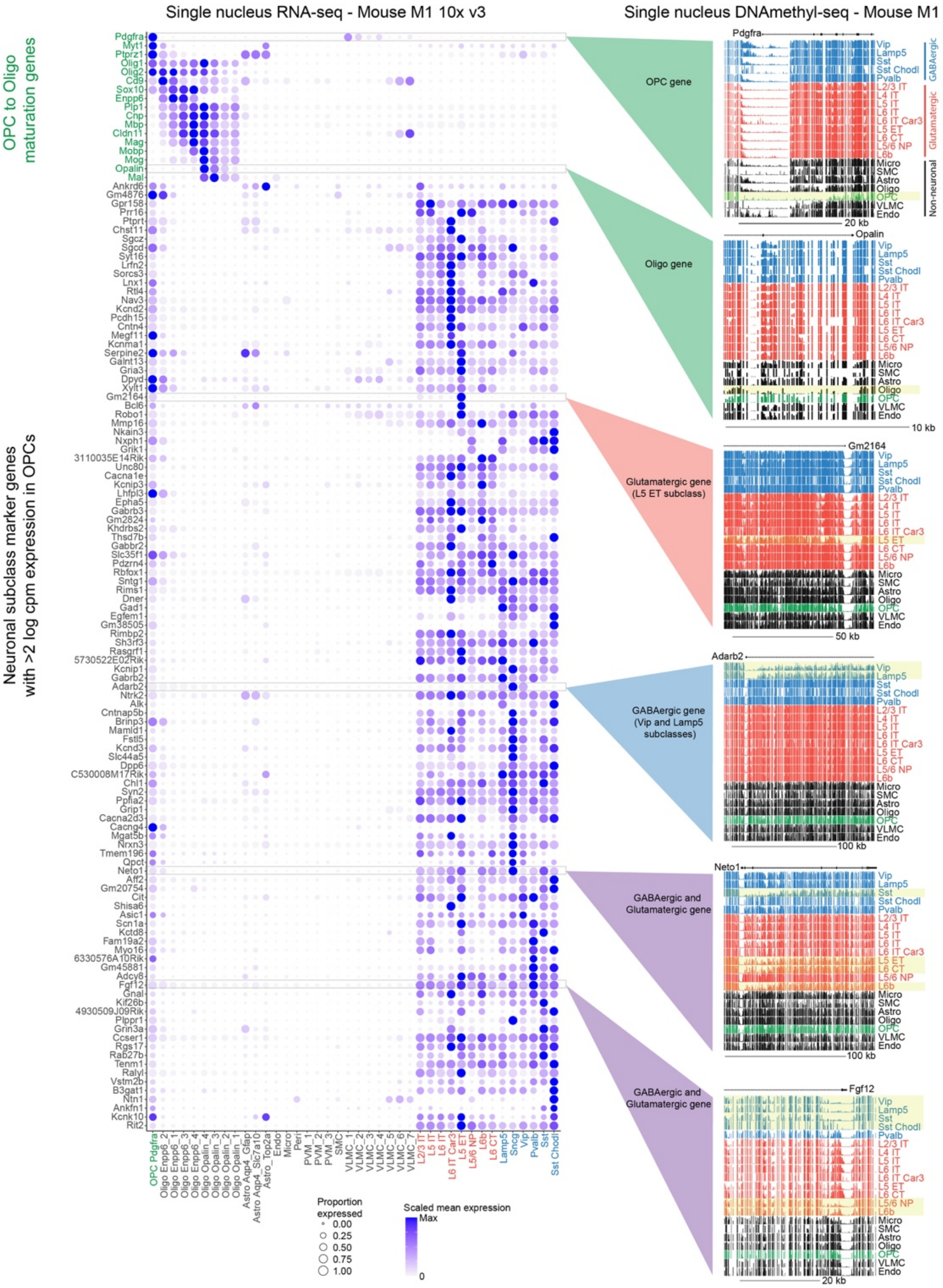

SI Fig. 10

Transcriptomic and epigenomic profiling of neuronal subclass genes in OPCs**.** Dot plot of OPC to oligodendrocyte maturation genes (green) and neuronal subclass marker genes for all non-neuronal clusters and neuronal subclasses in dataset. Expression is scaled by gene across all clusters and subclasses. Blue indicates GABAergic and red indicates glutamatergic neuronal subclasses. Clusters of gene body DNA hypomethylation are highlighted in yellow.

SI Movie Legends
See movies here: https://vimeo.com/user18027413/videos

SI Movie 1
Satellite Glia. All types of glia can be in the satellite position, in close apposition to neuronal cell somas. This movie shows both an OPC and a microglial cell together on the soma of a pyramidal neuron with their glial branches intertwined. https://vimeo.com/747877539

SI Movie 2
Following an OPC branch through the neuropil. This movie follows the branch of an OPC (Fig.2 *F)* back to its soma. Green ball shows the position in the branch in the insert. Many cellular organelles are present in the branch. https://vimeo.com/747877039

SI Movie 3
Axon engulfment. The tip of small collateral branch(gray) is engulfed within the cytoplasm of the OPC (pink). The axon tip remains attached the parent axon and that allows its identification as inhibitory. See SI Fig 5 *E-H*.
https://vimeo.com/747877154

SI Movie 4
Spine synapse engulfed by OPC. The dendritic spine in blue makes an excitatory synapse with the axon in gray. Both are still attached to their parent cell. There were few examples of whole synapses being ingested in this dataset. See Fig. 4*G-L*. https://vimeo.com/747877237

SI Material and Methods

Two-Photon Imaging

Before preparation for electron microscopy, mice underwent neurophysiology data acquisition conducted at Baylor College of Medicine (details in (1)). Note that this 2-photon data was not used in this study. Briefly, a 3 mm craniotomy was made centered on the primary visual cortex (V1; 2.7mm lateral of the midline, contacting the lambda suture), and the cortical window was then sealed with a 3 mm coverslip (Warner Instruments), using cyanoacrylate glue (VetBond). The mouse was allowed to recover for 1-2 hours prior to the imaging session. Imaging was performed in V1, in a 400 × 400 × 200 µm3 volume with the superficial surface of the volume at the border of L1 and L2/3, approximately 100um below the pia. Laser excitation was at 920nm at 25-45mW depending on depth. The objective used was a 25x Nikon objective with a numerical aperture of 1.1, and the imaging point-spread function was measured with 500 nm beads and was approximately 0.5 × 0.5 × 3 µm3 in x, y, and z. To aid in registration of optical physiology data to EM data, a wide field image of the cranial window visualizing the surface vasculature was provided in addition to a volumetric image stack of the vasculature, encompassing the region of tissue where the neurophysiology dataset was acquired. The vasculature was imaged by subcutaneous injection of 60 µL 2.5% Texas Red 3000MW Lysine fixable (Life Technologies D3328, allowing blood vessels and GCaMP6-expressing cell bodies to be imaged simultaneously by 2-photon microscopy. Mice were then transferred to the Allen Institute in Seattle and kept in a quarantine facility for 1 to 3 days, prior to perfusion.

Perfusion

After induction of anesthesia with isoflurane, the appropriate plane of anesthesia was checked by a lack of toe pinch reflex and the animals were transcardially perfused with 15 ml 0.15 M cacodylate buffer (EMS, Hatfield, PA, pH 7.4) followed by 30 ml fixative mixture containing 0.08 M cacodylate (pH 7.4), 2.5% paraformaldehyde (EMS), 1.25% glutaraldehyde (EMS) and 2 mM calcium chloride (Sigma). The perfusion solution was based on the work of ((2)Hua et al., 2015). Once the brain was removed it was placed into the same fixative solution to post-fix for 16 to 72 hours at 4 °C.

After perfusion of the animals and excision of the brain, the surface of the cortex was imaged using differential contrast lighting to visualize the surface vasculature of visual cortex and identify the region where the cranial window had previously been. This region was then prepared for Electron Microscopy. Details of the procedures to carefully map the neurophysiology site in the histological sections is described in (details in (1). We omitted these details here as no neurophysiology data was used in this manuscript, even though the anatomical data originates from the same block of tissue that was recorded with two-photon imaging. The brain was washed in CB (0.1 M cacodylate buffer pH 7.4) and embedded in 2% agarose. The agarose was trimmed and mounted for coronal sectioning in a Leica VT1000S vibratome; successive 200 µm thick slices were taken until the entire region of cortical tissue previously demarcated by manual markings was sectioned. During this procedure, we also acquired blockface images of each brain slice. The coronal sections containing the imaged site were then selected for histological processing (see below).

EM Histology

The histology protocol used here is based on the work of (2) and (3), with modifications to accommodate different tissue block sizes and to improve tissue contrast for transmission electron microscopy (TEM). Following several washes in CB (0.1 M cacodylate buffer pH 7.4), the vibratome slices were treated with a heavy metal staining protocol. Initial osmium fixation with 2% osmium tetroxide in CB for 90 minutes at room temperature was followed by immersion in 2.5% potassium ferricyanide in CB for 90 minutes at room temperature. After 2 x 30-minute washes with deionized (DI) water, the tissue was treated with freshly made and filtered 1% aqueous thiocarbohydrazide at 40 °C for 10 minutes. The samples were washed 2 x 30 minutes with DI water and treated again with 2% osmium tetroxide in water for 30 minutes

at room temperature. Double washes in DI water for 30 min each were followed by immersion in 1% aqueous uranyl acetate overnight at 4°. The next morning, the samples in the same solution were placed in a heat block to raise the temperature to 50° for 2 hours. The samples were washed twice in DI water for 30 minutes each, then incubated in Walton's lead aspartate pH 5.0 for 2 hours at 50 °C in the heat block. After double washes in DI water for 30 minutes each, the slices were dehydrated in an ascending ethanol series (50%, 70%, 90%, 3 x 100%) 10 minutes each and two transition fluid steps of 100 % acetonitrile for 20 minutes each. Infiltration with acetonitrile:resin dilutions at 2p:1p (24 h), 1p:1p (48 h) and 1p:2p (24 h) were performed on a gyratory shaker. Samples were placed in 100% resin for 24 hours, followed by embedment in Hard Plus resin (EMS, Hatfield, PA). The samples were cured in a 60 °C oven for 96 hours.

In order to evaluate the quality of samples during protocol development and before preparation for large scale sectioning, the following procedure was used for tissue mounting, sectioning and imaging. We evaluated each sample for membrane integrity, overall contrast and quality of ultrastructure. For general tissue evaluation, adjacent slices and tissue sections from the opposite hemisphere, processed in the same manner as the ROI slice, were cross-sectioned and thin sections were taken for evaluation of staining throughout the block neighboring the region of interest.

Ultrathin Sectioning

The tissue block was trimmed to contain the neurophysiology recording site which is the region of interest (ROI) then sectioned to 40 nm ultrathin sections. For both trimming and sectioning a Leica EM UC7 ultramicrotome was equipped with a diamond trimming tool and an Ultra 35 diamond knife (Diatome USA) respectively. Sectioning speed was set to 0.3 mm/sec. Eight to ten serial thin sections were cut to form a ribbon, after which the microtome thickness setting was changed to 0 nm in order to release the ribbon from the knife edge. Then, using an eyelash superglued to a handle, ribbons were organized to pairs and picked up as pairs to copper grids (Pelco, SynapTek, 1.5 mm slot hole) covered by 50nm thick LUXFilm support (Luxel Corp., Friday Harbor, WA).

Electron microscopy imaging

The imaging platform used for high throughput serial section imaging is a JEOL-1200EXII 120kV transmission electron microscope that has been modified with an extended column, a custom scintillator, and a large format sCMOS camera outfitted with a low distortion lens. The column extension and scintillator facilitate an estimated 10-fold magnification of the nominal field of view with negligible impact on resolution. Subsequent imaging of the scintillator with a high-resolution, large-format camera allows the capture of fields-of-view as large as 13x13 μm at 4 nm resolution. As with any magnification process, the electron density at the phosphor drops off as the column is extended. To mitigate the impact of reduced electron density on image quality (shot noise), a high-sensitivity sCMOS camera was selected and the scintillator composition tuned in order to generate high quality EM images within exposure times of 90 - 200 ms(4).

Image volume assembly and morphological segmentation

Aligning the individual image tiles and sections into a coherent three-dimensional volume and segmenting the cellular morphology for the P36 dataset was performed as previously described within(1, 5, 6).

Proofreading and Annotation of Volumetric Imagery Data

We used a combination of Neuroglancer (Maitin-Shepard, https://github.com/google/neuroglancer) and custom tools to annotate and store labeled spatial points(7). In brief, we used Neuroglancer to simultaneously visualize the imagery and segmentation of the 3D EM data. A custom branch of Neuroglancer was developed that could interface with a “dynamic” segmentation database, allowing users to correct errors (i.e., either merging or splitting neurons) in a centralized database from a web browser. Neuroglancer has some annotation functionality, allowing users to place simple annotations during a

session, but does not offer a way to store them in a central location for analysis. We thus built a custom cloud-based database system to store arbitrary annotation data centered associated with spatial points that could be propagated dynamically across proofreading events. Annotations were programmatically added to the database using a custom python client and, in relevant cases, after parsing temporary Neuroglancer session states using custom python scripts. These spatial points and their associated data (e.g., synapse type, cell body ID number, or cell types) were linked to stored snapshots of the proofreading for querying and reproducible data analysis. All data analyzed here came from the "v183" snapshot.

Visualization and Analysis of Mesh Data

Neuronal meshes were computed by Igneous (https://github.com/seung-lab/igneous) and kept up to date across proofreading. Meshes were analyzed in a custom python library, MeshParty (https://github.com/sdorkenw/MeshParty), that extends Trimesh (https://trimsh.org) with domain-specific features and VTK (https://www.vtk.org) integration for visualization. In cases where skeletons were used, we computed them with a custom modification of the TEASAR algorithm(8) on the vertex adjacency graph of the mesh object implemented as part of MeshParty. In order to associate annotations such as synapses or AIS boundary points with a mesh, we mapped point annotations to the closest mesh vertex after removing artifacts from the meshing process.

Quantification and Statistical Analysis

A t-test (two sample assuming unequal variance, two tailed) was performed in Microsoft Excel to compare number and densities of phagolysosomes.

Annotation of cells in the EM volume

We used a combination of Neuroglancer (Maitin-Shepard https://github.com/google/neuroglancer) and custom tools to annotate and store labeled spatial points. In brief, we used Neuroglancer to simultaneously visualize the imagery and segmentation of the 3D EM data. Neuroglancer incorporates the capability to store and remark on xyz points within the data. These spatial points and their associated data (e.g., Cell type, cell body ID number, or organelles) were linked to stored spreadsheets and documents.

Mouse primary OPC culture

Cerebral cortices from post-natal day 6 or 7 (P6/P7) CD1 mouse pups (Charles River) were collected, and tissue was dissociated using MACS Milltenyi Biotec Neural Tissue Dissociation Kit (P) (130-092-628), according to the manufacturer's instructions. Briefly, cortices were collected from each pup and incubated at 37˚C with gentle rotation using a MACSmix™ Tube Rotator (130-090-753) following digestion with pre-warmed enzyme P mix. Cells were then mechanically dissociated and passed through a 70µm MACS SmartStrainer and a 70µm Pre-Separation Filter to remove cell clumps before incubation with Anti-O4 Microbeads (130-094-543). Cells were then loaded into an MS column (130-042-201) in for magnetic separation, and positively-selected cells were collected following extrusion through the column.

Cells were plated at a density of about 20,000 cells / well in a 24-well plate with 1.5mm coverslips in each well that had previously been coated overnight with poly-d-lysine (50µL PDL in 250mL dH_2O). Cells were expanded overnight at 37˚C in OPC media containing recombinant human PDGF-AA and Neurotrophin-3 until optimal density was reached. The plates were then processed for immunofluorescence staining.

Staining of primary OPC cultures

Coverslips were washed once with 1x PBS before 4% PFA was applied for 13 minutes at room temperature. Cells were then washed twice with 1x PBS (5 minutes/wash) before blocking in 0.5% Saponin, 5% normal donkey serum, and 1x PBS) for 1 hour at room temperature. Cells were then stained with primary antibodies against NG2 proteoglycan (guinea pig, 1:100, custom Bergles antibody), LAMP2

(rat, Abcam, 1:200) to visualize OPC cell bodies/ processes and lysosomes, respectively. Following overnight incubation at 4˚C with rotation, cells were washed three times with 1x PBS (5 minutes/wash) at room temperature. AlexaFluor secondary antibodies (488 donkey anti-rat and 647 donkey anti-guinea pig) were applied for 1 hour at room temperature in the dark. Cells were again washed three times, DAPI stain was applied for 10 minutes, and cells were washed again twice before coverslips were removed and mounted to slides using ProLong^{TM} Gold Antifade Mountant and allowed to dry at room temperature for 24 hours.

Imaging and analysis of primary OPC cultures
Cells from three separate culture experiments were used for the analysis. All images were taken at 63x magnification using the AiryScan function on a Zeiss 710 confocal microscope. Airyscan-processed z-stacks of NG2^{+} individual OPCs were projected using the maximum intensity projection function, and the number of LAMP2^{+} vesicles within cell processes were quantified.

Reagents used in primary OPC cultures
OPC Culture Tools **– all Miltenyi** (Cat no.)
MACS Milltenyi Biotec Neural Tissue Dissociation Kit (P) (130-092-628)
MACS Milltenyi Biotec Anti-O4 MicroBeads (130-094-543)
MACS SmartStrainer (70µm) (130-090-753)
MACSmix^{TM} Tube Rotator (130-090-753)
Pre-Separation Filters, 70µm (130-095-823)
MACS MS Columns (130-042-201)
MACS Multistand (130-042-303)
MiniMACS^{TM} Separator (130-042-102)

Antibodies
Anti-NG2 (Guinea pig, source: Bergles Laboratory)
Anti-LAMP2 (Abcam, cat no. ab13524)

Additional Reagent
0.5% saponin (Millipore Sigma, 47036)
ProLong^{TM} Gold Antifade Mountant (ThermoFisher, cat no. P10144)

Experimental Models: Organisms/ Strains
CD1 mice, Charles River (Strain number 022)

Molecular analysis of OPCs and microglia with single nucleus RNA-seq and single nucleus DNA methyl-seq2 dataset.
We analyzed recently described Chromium 10x V3 single nucleus RNA-seq and single nucleus DNA methyl-seq2 datasets from mouse primary motor cortex(9), available from the Neuroscience Multi-omic Data Archive (NeMO, https://assets.nemoarchive.org/dat-ch1nqb7). Only the 159,738 nuclei from the dataset generated by the Broad Institute were used.
For gene expression dot plots, the mean UMIs per cluster and proportion of cluster with UMIs greater than 1 were calculated using the scrattch.hicat v0.0.22 library (https://github.com/AllenInstitute/scrattch.hicat) and visualized using ggplots2 v3.3.3 library(10) in R v3.4.1. (10) in R v3.4.1. Mean expression was scaled from 0 to 1 for each gene for each major cell class (i.e. non-neuronal and neurons). To find neuronal subclass marker genes, we first created a Seurat (11), (12) object of only the neuronal cell types, downsampled to 500 nuclei per neuronal subclass. (11), (12) object of only the neuronal cell types, downsampled to 500 nuclei per neuronal subclass. We then used the FindAllMarkers function from Seurat v3.2.0 with the 'roc' test to identify differentially expressed genes that were enriched in a particular neuronal subclass compared to other neurons. Neuronal subclass

marker genes were then filtered to include genes with greater than 2 log2FC in OPCs and microglia relative to each mature oligodendrocyte cluster (Oligo Opalin_1-3), and with greater than 2 log2 cpm expression in OPCs and microglia.
To visualize single nucleus DNA methyl-seq2 tracks, we loaded gene body methylation (CGN) tracks into the UCSC genome browser. Example tracks of neuronal subclass marker genes that showed
marker genes were then filtered to include genes with greater than 2 log2FC in OPCs and microglia relative to each mature oligodendrocyte cluster (Oligo Opalin_1-3), and with greater than 2 log2 cpm expression in OPCs and microglia.
To visualize single nucleus DNA methyl-seq2 tracks, we loaded gene body methylation (CGN) tracks into the UCSC genome browser. Example tracks of neuronal subclass marker genes that showed
expression in OPCs were identified to highlight the lack of DNA hypomethylation in OPCs.

Specific GO term gene lists for lysosome, phagocytosis, and synapse assembly were downloaded from http://www.informatics.jax.org/go/term/ on January 20th, 2021(13),(14, 15).. Each gene list was filtered to include genes with greater than 2 log2FC in OPCs and/or microglia relative to each mature oligodendrocyte cluster, and with greater than 2 log2 cpm expression in OPCs and/or microglia.

Data availability
The raw images, segmentation, and synaptic connectivity will be made available upon or before publication.
Code availability
All software is open source and available at http://github.com/seung-lab if not otherwise mentioned.
Alembic: Stitching and alignment.
CloudVolume: Reading and writing volumetric data, meshes, and skeletons to and from the cloud
Chunkflow: Running convolutional nets on large datasets
DeepEM: Training convolutional nets to detect neuronal boundaries.
DynamicAnnotationFramework: Proofreading and connectome updates (visit https://github.com/seung-lab/AnnotationPipelineOverview for repository list)
Igneous: Coordinating downsampling, meshing, and data management.
MeshParty: Interaction with meshes and mesh-based skeletonization (https://github.com/sdorkenw/MeshParty)
MMAAPP: Watershed, size-dependent single linkage clustering, and mean affinity agglomeration.
PyTorchUtils: Training convolutional nets for synapse detection and partner assignment (https://github.com/nicholasturner1/PyTorchUtils).
Synaptor: Processing output of the convolutional net for predicting synaptic clefts (https://github.com/nicholasturner1/Synaptor).
TinyBrain and zmesh: Downsampling and meshing (precursors of the libraries that were used).

References

1. S. Dorkenwald *et al.*, Binary and analog variation of synapses between cortical pyramidal neurons
. *bioRxiv* 10.1101/2019.12.29.890319 (2019).
2. Y. Hua, P. Laserstein, M. Helmstaedter, Large-volume en-bloc staining for electron microscopy-based connectomics. *Nat Commun* **6**, 7923 (2015).
3. J. C. Tapia *et al.*, High-contrast en bloc staining of neuronal tissue for field emission scanning electron microscopy. *Nat Protoc* **7**, 193-206 (2012).
4. W. Yin *et al.*, A petascale automated imaging pipeline for mapping neuronal circuits with high-throughput transmission electron microscopy. *Nat Commun* **11**, 4949 (2020).
5. C. M. Schneider-Mizell *et al.*, Structure and function of axo-axonic inhibition. *Elife* **185**, 1082-1100 (2021).
6. N. L. Turner *et al.*, Multiscale and multimodal reconstruction of cortical structure and function. *bioRxiv* 10.1101/2020.10.14.338681 (2020).
7. J. Wu, W. M. Silversmith, K. Lee, H. S. Seung, Chunkflow: hybrid cloud processing of large 3D images by convolutional nets. *Nature Methods* **18**, 328-330 (2021).
8. I. B. M. Sato, M. A. Bender, A. E. Kaufman and M. Nakajima, TEASAR- Tree-structure Extraction Algorithm for Accurate and Robust Skeletons *Proceedings the Eighth Pacific Conference on Computer Graphics and Applications* 10.1109/PCCGA.2000.883951, 281-449 (2000).
9. Z. Yao *et al.*, A taxonomy of transcriptomic cell types across the isocortex and hippocampal formation. *Cell* **184**, 3222-3241 e3226 (2021).
10. H. Wickham, G. Grolemund, *R for Data Science: Import, Tidy, Transform, Visualize, and Model Data* (O'Reilly Media, 2016).
11. T. Stuart *et al.*, Comprehensive Integration of Single-Cell Data. *Cell* **177**, 1888-1902 e1821 (2019).
12. A. Butler, P. Hoffman, P. Smibert, E. Papalexi, R. Satija, Integrating single-cell transcriptomic data across different conditions, technologies, and species. *Nat Biotechnol* **36**, 411-420 (2018).
13. C. J. Bult *et al.*, Mouse Genome Database (MGD) 2019. *Nucleic Acids Res* **47**, D801-D806 (2019).
14. C. M. Smith *et al.*, The mouse Gene Expression Database (GXD): 2019 update. *Nucleic Acids Res* **47**, D774-D779 (2019).
15. D. M. Krupke *et al.*, The Mouse Tumor Biology Database: A Comprehensive Resource for Mouse Models of Human Cancer. *Cancer Res* **77**, e67-e70 (2017).
